# Cocirculation of Swine H1N1 Influenza A Virus Lineages in Germany

**DOI:** 10.3390/v12070762

**Published:** 2020-07-15

**Authors:** Roland Zell, Marco Groth, Andi Krumbholz, Jeannette Lange, Anja Philipps, Ralf Dürrwald

**Affiliations:** 1 Section of Experimental Virology, Institute for Medical Microbiology, Jena University Hospital, Friedrich Schiller University Jena, D-07745 Jena, Germany; krumbholz@infmed.uni-kiel.de (A.K.); jeannette.lange@pei.de (J.L.); anja.philipps@thermofisher.com (A.P.); duerrwaldr@rki.de (R.D.); 2 CF DNA Sequencing, Leibniz Institute on Aging, Fritz Lipmann Institute, D-07745 Jena, Germany; marco.groth@leibniz-fli.de; 3 Institute of Infection Medicine, Kiel University and University Medical Center Schleswig-Holstein, D-24105 Kiel, Germany; 4 Paul-Ehrlich-Institut, D-63225 Langen, Germany; 5 Thermo Fisher Scientific GENEART GmbH, D-93059 Regensburg, Germany; 6 Robert Koch Institute, D-13353 Berlin, Germany

**Keywords:** swine influenza virus, reassortment, H1N1, antigenic drift, phylogenetic analysis, Germany

## Abstract

The genome analysis of 328 H1N1 swine influenza virus isolates collected in a 13-year long-term swine influenza surveillance in Germany is reported. Viral genomes were sequenced with the Illumina next-generation sequencing technique and conventional Sanger methods. Phylogenetic analyses were conducted with Bayesian tree inference. The results indicate continued prevalence of Eurasian avian swine H1N1 but also emergence of a novel H1N1 reassortant, named Schneiderkrug/2013-like swine H1N1, with human-like hemagglutinin and avian-like neuraminidase and internal genes. Additionally, the evolution of an antigenic drift variant of A (H1N1) pdm09 was observed, named Wachtum/2014-like swine H1N1. Both variants were first isolated in northwest Germany, spread to neighboring German states and reached greater proportions of the H1N1 isolates of 2014 and 2015. The upsurge of Wachtum/2014-like swine H1N1 is of interest as this is the first documented persistent swine-to-swine spread of A (H1N1) pdm09 in Germany associated with antigenic variation. Present enzootic swine influenza viruses in Germany now include two or more co-circulating, antigenically variant viruses of each of the subtypes, H1N1 and H1N2.

## 1. Introduction

Influenza A viruses (IAV) of the family *Orthomyxoviridae* infect a wide range of hosts including feral water fowl (main hosts), poultry and several mammalian species (e.g., humans, pigs and horses). Swine influenza is of particular significance due to its great economic importance. Globally, there are three areas with major swine production, each with characteristic, enzootic swine influenza A virus (S-IAV) strains. These areas are Europe, North America and East/Southeast Asia. Furthermore, pig holding is increasing in Central and South America which probably also contributed to the emergence of A (H1N1) pdm09 [1].

Eurasian avian-like swine H1N1 (EA swH1N1) emerged in Belgium and Germany in 1979 [2,3] after transspecies infection of pigs with an avian IAV. This virus achieved sustained circulation in pigs and spread to many European countries but also to China and Korea [4,5]. Analysis of German avian IAV isolates which had been collected in the 1970s suggested that EA swH1N1 is a triple reassortant with a unique internal gene cassette (IGC) that had not been detected in avian IAVs before [6]. Later, EA swH1N1 reassorted with seasonal H3N2 and seasonal H1N1 to yield so-called human-like (hu-like) swH3N2 and hu-like swH1N2 (reviewed in [4]). Recent investigations of European S-IAV isolates revealed different lineages of swH1N2 and swH3N2 reassortants on the continent and the UK [7,8]. All lineages have the EA swH1N1 internal gene cassette (EA IGC), but hemagglutinin (HA) and neuraminidase (NA) genes are either A/Port Chalmers/1/1973-like (H3N2), A/Albany/20/1974-like (H3N2) or Singapore/6/1986-like (H1N1). Another independent hu-like swH1N2 reassortant came up in Italy [9]. This 7 + 1 reassortant has an A/Singapore/6/1986-like (H1N1) HA gene, an A/Panama/2007/1999-like (H3N2) NA gene and the EA IGC. After the emergence of A (H1N1) pdm2009 (H1_pdm_N1_pdm_), numerous anthroponotic infections of pigs have been detected in many European countries both at the ongoing pandemic and thereafter [7,10,11,12,13,14]. Due to significant cross-reaction of antibodies directed against EA swH1N1 [15], H1_pdm_N1_pdm_ failed to establish stable infection chains in pigs in Germany and other countries with high pig density and prevalence of EA swH1N1. However, numerous reassortants arose of which the Papenburg/2010-like swH1N2 viruses, a stable 7 + 1 swH1N2 reassortant with HA and IGC of H1_pdm_N1_pdm_ and NA gene of the contintental Gent/1984-like swH3N2, became endemic in Germany, Belgium and the Netherlands [7,8,13,16].

Losses caused by swine influenza are economically significant. Therefore, swine influenza surveillance is of increasing importance. Recently, the ESNIP3 consortium reported results on a S-IAV survey conducted 2010–2013 in 14 European countries [17]. Independently, members of the German FluResearchNet consortium have conducted a 13-year long-term swine influenza surveillance in Germany, 2003–2015, which enabled the isolation of 1310 S-IAV isolates. The sequencing results of 267 swH1N2 isolates have been published recently [8]. The data indicated (i) the replacement of the continental Gent/1999-like swH1N2 by the Diepholz/2008-like, Emmelsbuell/2009-like and Papenburg/2010-like swH1N2 reassortants, and (ii) the emergence of other swH1N2 reassortants that were partly persistent (Gladau/2012-like swH1N2), partly ephemeric.

Here, we report on the genome analysis of 328 swH1N1 isolates, mostly from Germany. The present results demonstrate continued prevalence of EA swH1N1 but co-circulation with:

- A H1_pdm_N1_pdm_ variant which emerged in 2014 and lacks cross-reactivity with EA swH1N1 and H1_pdm_N1_pdm_, and
- A novel swH1N1 reassortant with the HA gene of the new prevalent Diepholz/2008-like swH1N2 lineage.

## 2. Materials and Methods

### 2.1. Study Design

The study design has been described previously (for details see [8]). Briefly, for a passive swine influenza survey, 8122 samples (7798 nasal swabs, 165 bronchoalveolar lavages, 159 lung tissues) of diseased pigs were sent in by veterinarians from 14 of the 16 German states. A few additional samples were from Austria, Switzerland and the Netherlands. All samples were analyzed by S-IAV-specific RT-PCR and virus isolation. Of 1310 virus isolates 810 were selected for sequencing, 327 of which were of the H1N1 subtype. In addition, 21 H1N1 isolates collected between 1979 and 2001 were included in the molecular analysis, of which the genomes of 16 had been published previously [6,13,18]. All in all, 348 H1N1 S-IAV genomes were analyzed, 328 of which are new S-IAV genomes. Supplementary Table S1 summarizes GenBank accession numbers, country and sampling dates of all S-IAV H1N1 strains sequenced by the Jena Swine Influenza Virus Sequencing Initiative (SIVSI).

### 2.2. Cell Lines, Virus Isolation and Virus Amplification

Madin-Darby bovine kidney cells (MDBK, ATCC CCL-22) and Madin-Darby canine kidney cells (MDCK, ATCC CCL-34) were maintained in Dulbecco’s modified MEM (DMEM) supplemented with 10% fetal bovine serum, 100 U/mL penicillin and 100 µg/mL streptomycin. For virus isolation, PCR-positive samples were used for inoculation of embryonated hen eggs and MDBK cells. All virus isolates were typed by hemagglutination inhibition assays and RT-PCR as described in [17,19]. Virus was propagated in MDCK/MDBK cells using serum-free DMEM with 3 µg/mL trypsin and 25 mM MgCl_2_; cells were infected at a multiplicity of infection of 0.01. After 2–3 days, supernatants were centrifuged at 1000× *g* and aliquoted. Virus stocks were stored at −80 °C until use.

### 2.3. Nucleic Acid Extraction and PCR Analysis

For RNA extraction following kits were used: QIAamp Viral RNA Mini Kit (Qiagen, Hilden, Germany) and Chemagic Viral DNA/RNA Kit (PerkinElmer, Waltham, MS, USA). For diagnostics, reverse transcription was performed with the Superscript One step RT-PCR kit (Invitrogen). The following oligonucleotide primers were used for PCR diagnostics: M: FLU-M52F: 5′-CTTCTAACCGAGGTCGAAACG-3′, FLU-M253R: 5′-AGGGCATTTTGGACAAAKCGTCTA-3′ [19]; H1 (H1N1): H1-550F: 5′-AACAAYAARGRGAAAGAAGT-3′ H1-1016R: 5′-GGGACDTTYCTTARTCCTGT-3′; H1 (H1N2): 18F: 5′-AACAATAGAGAAGAAGAAGT-3′, 7R: 5′-GGAATGTTCCTTAGTCCTGT-3′; panH1N1: pH1-F238: 5′-GGAAATCCAGAGTGTGAATCACTC-3′, pH1-R463: 5′-GAGGACATGCTGCCGTTACACC-3′; N1: NAN-1F: 5′-CGATGGACCAAGTAATGGGC-3′, NAN-1R: 5′-AATGGCAACTCAGCACCGTC-3′; conditions: 30 min at 45 °C (1×), 15 min at 95 °C (1×), 0.5 min at 94 °C, 0.5 min at 48 °C, 1 min at 68 °C (35×), 10 min at 68 °C (1×), 0.016 min at 20 °C (1×), thereafter at 4 °C.

### 2.4. Sequence Analysis

Plaque-purified swH1N1 isolates were used for RNA extraction and subsequent conventional Sanger sequencing or for Illumina sequencing as described previously [13,20]. Total RNA was prepared employing the Qiagen RNeasy Mini Kit (Qiagen, Hilden, Germany). For cDNA synthesis, 5 µg of total RNA were reverse transcribed utilizing the universal influenza virus primer (5′-RGCRAAAGCAGG-3′) [21] and 20 µL RevertAid premium reverse transcriptase solution (ThermoFisher Scientific, St. Leon-Rot, Germany) following the manufacturer’s protocol. Then, segment-specific amplification was conducted with published oligonucleotide primers [22]. PCR products were purified and sequenced employing the CEQ DTCS Quick Start Kit (Beckman Coulter, Krefeld, Germany).

Methods of Illumina sequencing of IAV and de novo assembly of virus genomes has been described in [13], except that sequencing was run on another platform (GenomeAnalyzer IIx or HiSeq2000/2500). Consensus sequences were determined by mapping the reads to reference genomes as described in [23]. Assignment to IAV lineages was based on the assembled contigs. All sequences were submitted to GenBank (accession numbers MK364805-MK367387; compare Supplementary Table S1).

### 2.5. Phylogenetic Analyses

Only complete open reading frames (HA-ORF: 1707 nt; NA-ORF: 1413 nt; PB2-ORF: 2280 nt; PB1-ORF: 2274 nt; PA-ORF: 2151 nt; NP-ORF: 1497 nt; M1/M2-encoding gene region: 982 nt; NS1/NS2-encoding gene region: 835 nt) were used for phylogenetic analyses. Additional sequences were retrieved from public databases (GenBank, GISAID) and aligned with MEGA5.2 [24]. For alignments, 950 HAH1 sequences, 577 NAN1 sequences and 258 HAH1_pdm_ sequences were compiled depending on the availability of sequence data. Two coalescent tree inference methods (MrBayes, BEAST) were used for phylogenetic analyses [25,26] applying optimal substitution models on the basis of the Bayesian information criterion and the corrected Akaike information criterion. The substitution model was selected with the find-best-model option implemented in MEGA5.2 and Mega X [24,27].

### 2.6. Nomenclature of S-IAV Lineages and Clades

Designations of S-IAV lineages which are established in literature like European/Eurasian avian (EA) swH1N1, human-like (hu-like) swH1N2 and hu-like swH3N2 were retained. For subgrouping of the HAH1 gene, the lineage and sublineage designations of Anderson et al. [28] were adopted (hereafter referred to as Anderson-2016 nomenclature). Reassortant Diepholz/2008-like, Emmelsbuell/2009-like, Papenburg/2010-like, Gladau/2012-like swH1N2 viruses have been introduced recently [8]. The novel viruses described here were named after a representative isolate, Schneiderkrug/2013-like swH1N1 and Wachtum/2014-like swH1_pdm_N1_pdm_. The prefix sw (swine) indicates the porcine host. Seasonal human H1N1 and A(H1N1)pdm2009 virus were distinguished by adding the subscripts ‘seas’ and ‘pdm’ to type designations (H1_seas_N1_seas_, H1_pdm_N1_pdm_).

### 2.7. Antigenic Analysis

Hemagglutination inhibition (HI) assays were conducted as described [20]. Briefly, all sera were pre-treated with neuraminidase (Sigma, EC3.2.1.18 Type IV from *Clostridium perfringens*, 14–18 h at 37 °C); then sodium citrate (1.5%) was added and inactivation was carried out for 30 min at 56 °C, followed by adsorption to chicken erythrocytes for 1 h at 4–8 °C. Antigens were adjusted to eight hemagglutinating units (HU). To carry out the test, the sera (already pre-diluted 1:10 through the pretreatment) were titrated in microtitre plates (log2). The same volume of antigen suspension (25 µL) adjusted to 8 HU was pipetted into each of the wells of the microtitre plate and the mixture incubated for 30 min at room temperature. Then, 50 µL of a 0.5% chicken erythrocyte suspension was added and the plates were allowed to stand for 30 min at room temperature.

Hyperimmune and immune sera in pigs were established and hemagglutination inhibition assays (HI) were performed as described previously [13,15]. In short, immune sera were generated by twofold immunization of 10 pigs with inactivated and Carbopol-adjuvanted virus of each subtype (EA swH1N1: A/sw/Harlebach/2998/2004; H1_pdm_N1_pdm_: A/Jena/VI2688/2010; swH1_pdm_N2: A/sw/Papenburg/12653/2010; swH1_pdm_N1_pdm_: A/sw/Wachtum/20657/2014) within 21 days; blood was taken 10 days after second immunization. The sera were pooled and tested against the virus isolates by HI. Hyperimmune sera were established by four-fold immunization (0, 14, 28 and 54 days after first shot) of one pig each (intramuscularly) with 64 hemagglutinating units of the corresponding inactivated virus adjuvanted with Freund’s adjuvant (Sigma-Aldrich) or mineral oil (ISA25, Seppic). Blood samples were taken 70 days after first administration of the immunogen. Animal experiments were approved by the Landesverwaltungsamt Sachsen-Anhalt (Az 42502-3-401, 42502-3-642Ä, 42502-3-743, 45502-3-579).

## 3. Results

**Figure 1:**
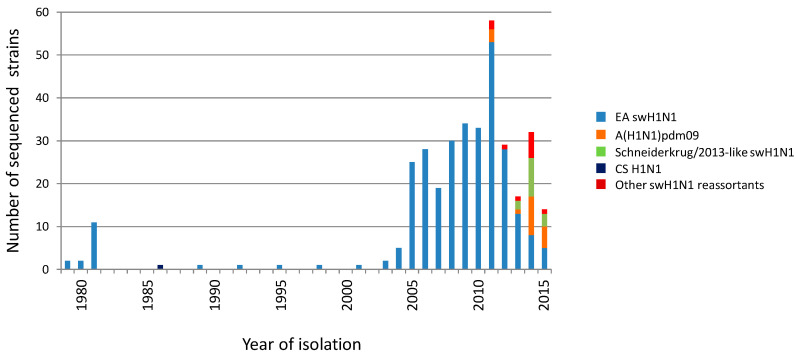
Distribution of swH1N1 isolates. Year of isolation of H1N1 strains sequenced in this study. Different clades are indicated.

The German swine influenza surveillance, 2003–2015, was conducted by members of the FluResearchNet consortium and succeeded to collect 1310 S-IAV isolates. Our S-IAV archive was completed by 40 virus strains of other sources from 1979 to 2001. These 40 virus strains plus 810 isolates of the surveillance have been sequenced by the Jena Swine Influenza Virus Sequencing Initiative, i.e., 348 swH1N1, 267 swH1N2, 229 swH3N2 and six swH3N1 isolates [8]. Genetic analysis of 348 swH1N1 isolates (328 of which were unpublished) revealed 304 EA swH1N1 strains, 18 H1_pdm_N1_pdm_ strains, 11 reassortants with gene segment(s) of H1_pdm_N1_pdm_, 14 isolates of the novel Schneiderkrug/2013-like swH1N1 reassortant and one classical swine (CS) H1N1 (Figure 1).

### 3.1. Phylogenetic Analysis of German swH1N1 Isolates

**Figure 2:**
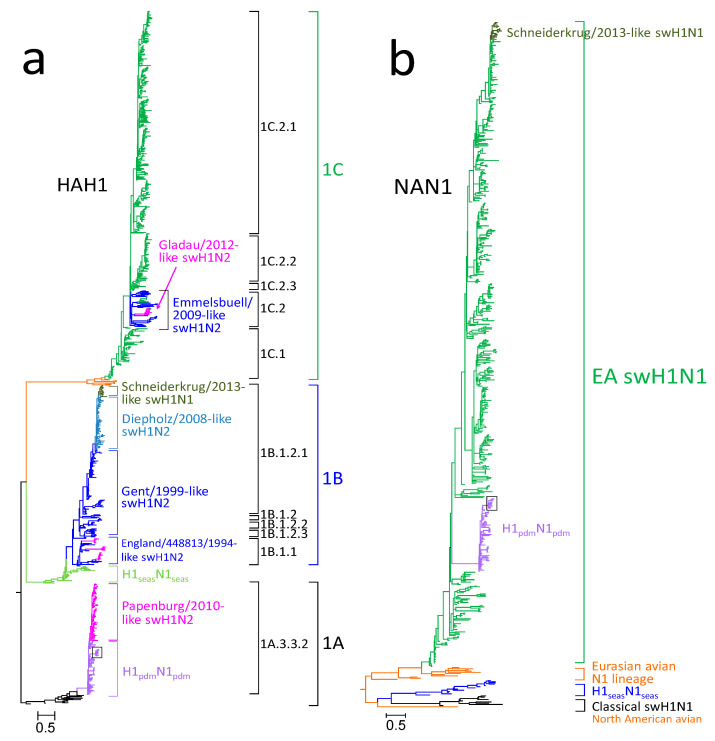
Phylogenetic analyses of hemagglutinin (HA) and neuraminidase (NA) genes using Bayesian tree inference. Relevant lineages and sublineages are indicated. The scale bars indicate substitutions per site. The Wachtum/2014-like swH1_pdm_N1_pdm_ are boxed. (**a**) Analysis of 950 HAH1 sequences of lineages 1A, 1B and 1C (Anderson-2016 nomenclature). Details are presented in Supplementary Figure S1. (**b**) Analysis of 577 NAN1 sequences. Details are presented in Supplementary Figure S2. Color code: green, EA swH1N1; light green, seasonal H1N1; olive, Schneiderkrug/2013-like swH1N1; purple, H1_pdm_N1_pdm_; magenta, H1_pdm_N2 reassortants; blue, swH1N2; light blue, Diepholz/2008-like swH1N2; black, classical swine H1N1; ochre, avian H1.

**Figure 3:**
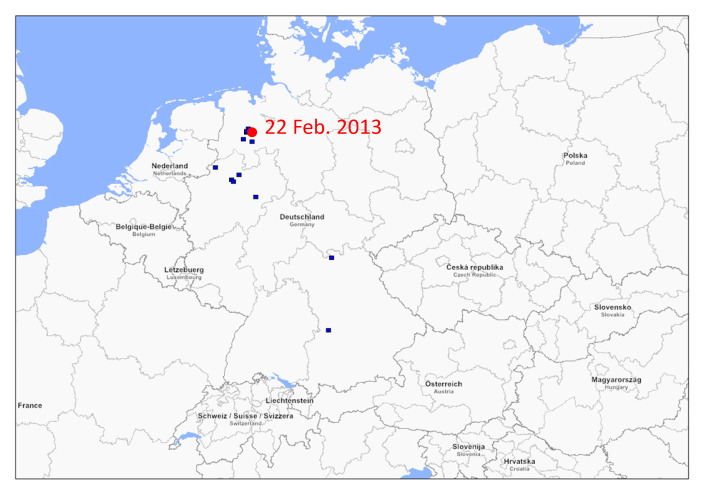
Distribution of Schneiderkrug/2013-like swH1N1 in Germany. Place and collection date of the first isolate is indicated.

Phylogenetic analysis revealed that the HA gene of German swH1N1 isolates belongs to three co-circulating HAH1 lineages, named 1A, 1B and 1C according to the Anderson-2016 nomenclature (Figure 2a, Supplementary Figure S1). The HA gene of the prevalent EA swH1N1 strains belongs to lineage 1C, whereas the HA of the H1_pdm_N1_pdm_ belongs to lineage 1A and the Schneiderkrug/2013-like swH1N1 to lineage 1B. The latter viruses emerged in February 2013 in northwest Germany and have been detected in three German States (Figure 3). The viruses persisted until the end of the surveillance in December 2015. Five sublineages of 1C have been defined by Anderson et al. on basis of phylogenetic clustering: (i) Sublineage 1C.1 comprises the continental swH1N1 isolates of 1979–2000 and all English isolates, 1992–2011. (ii) Sublineage 1C.2 includes a few Danish and German swH1N1 strains, 1993–2013, plus the Emmelsbuell/2009-like swH1N2 and the Gladau/2012-like swH1N2. (iii) Sublineage 1C.2.1 contains swH1N1 strains of 2005–2015, (iv) sublineage 1C.2.2 strains of 2001–2014, and (v) sublineage 1C.2.3 five strains of 1999–2004. Beside phylogenetic clustering, no biological, geographical or other correlates could be identified that supported sub-classification of the S-IAV HA 1C lineage.

S-IAV strains with HA of lineage 1B are derivatives of H1_seas_N1_seas_, which disappeared after the 2009 pandemic. The swH1N2 strains of lineage 1B have been described recently [8]. Sublineage 1B.1.2.1 includes the continental swH1N2 (Gent/1999-like swH1N2) and two reassortants, the Diepholz/2008-like swH1N2 and the Schneiderkrug/2013-like swH1N1. Six segments of the latter reassortant (HA, PB1, PB2, PA, NP, M) are derived from the Diepholz/2008-like swH1N2, whereas the NA and NS segments originated from EA swH1N1.

**Figure 4:**
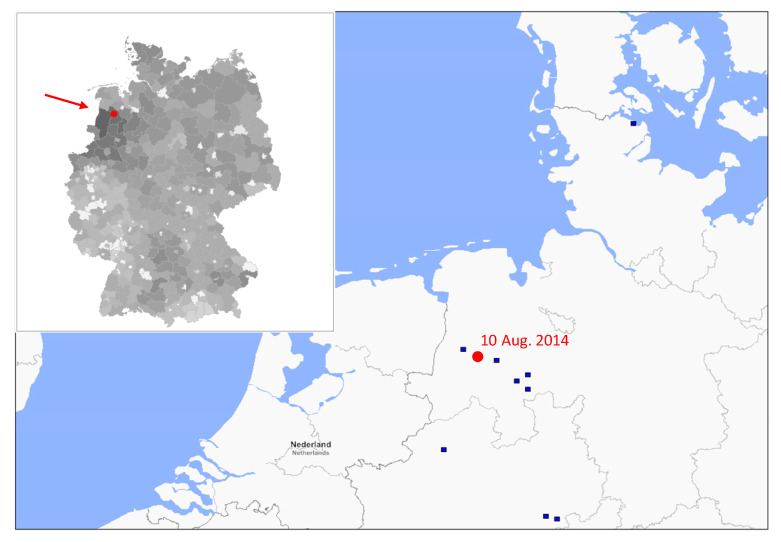
Incursion of Wachtum/2014-like swH1_pdm_N1_pdm_ into the German pig population. Dark squares indicate the places where isolates were collected; the place and sampling date of the first German isolate is printed in red. The inset presents the pig population density in Germany (gray shades correspond to the numbers of pigs per administrative district). The data were retrieved from the 2017-yearbooks of the 16 State Statistical Offices in Germany.

Lineage 1A includes H1_pdm_N1_pdm_ and derived H1_pdm_N2 strains (sublineage 1A.3.3.2). Whereas incursion of H1_pdm_N1_pdm_ into pigs has been observed several times since 2009 and persistent intra-herd transmission of S-IAV is a well-known phenomenon [29,30], farm-to-farm spread of H1_pdm_N1_pdm_ was not detected in the first years post-pandemic in Germany. In contrast, the Papenburg/2010-like swH1N2 soon became enzootic in Germany [13]. This situation changed in 2014, as the HA tree exhibits a cluster of 10 German sequences with greater 0.5 substitutions/site, which is exceptional (Figure 2a and Supplementary Figure S1). This result was also observed in the NA tree (Figure 2b and Supplementary Figure S2). This finding was confirmed in a refined phylogenetic analysis of HA which comprised 92 human H1_pdm_N1_pdm_ strains, 67 swH1_pdm_N1_pdm_, 86 swH1_pdm_N2 and 13 reference sequences (CS H1N1 and North American triple reassortant H1N1) (Supplementary Figure S3). This group of German swH1_pdm_N1_pdm_ was named Wachtum/2014-like viruses after its first isolate and belonged to genogroup 6 according to WHO nomenclature (September 2018 interim report of the Worldwide Influenza Centre; https://www.crick.ac.uk/partnerships/worldwide-influenza-centre/annual-and-interim-reports, accessed 12 September 2019). These viruses exhibited up to 6% diversity (*p*-distance) to other human and porcine H1_pdm_N1_pdm_ strains (data not shown). The first isolate emerged in August 2014 in Lower Saxony in northwest Germany and spread to the neighboring States Northrhine Westphalia and Schleswig-Holstein (Figure 4) but also to Italy (Supplementary Figure S3). In addition, one H1N2 reassortant with a Wachtum/2014-like HA gene was also isolated.

### 3.2. Analysis of Antigenic Sites of swH1N1

For analysis of the antigenic sites as defined by Caton and Brownlee [31] and Brownlee and Fodor [32], we compared aa 183-187/220-222/252-254 (site Ca1), aa 154-159/238-239 (site Ca2), aa 87-92 (site Cb), aa 141-142/170-181 (site Sa) and aa 201-212 (site Sb) of lineages 1A, 1B and 1C (Anderson-2016 nomenclature; see Supplementary Figure S5A–C).

Phylogenetic analysis demonstrates that the NA gene of all EA swH1N1, H1_pdm_N1_pdm_ and Schneiderkrug/2013-like swH1N1 belong to the same clade of the Eurasian avian N1 lineage, which emerged in 1979 (compare Figure 2b and Supplementary Figure S2). Comparison of the HAH1 and NAN1 trees, however, revealed traces of previous intratypic reassortment events (see Supplementary Figure S4). We have identified 15 sequence clusters which persisted between six and 21 years. Several HA clusters (e.g., #4, #8, #9, #10) are scattered in the NA tree, which is compatible with the concept of intratypic reassortment. Intratypic and intertypic reassortment is also evident from the phylogenetic trees of the internal genes (data not shown).

The H1_pdm_N1_pdm_ strains have been assigned into genogroups by the Worldwide Influenza Centre–WHO Collaborating Centre for Reference and Research on Influenza—The Francis Crick Institute on basis of partial HA sequences and their antigenic properties (https://www.crick.ac.uk/partnerships/worldwide-influenza-centre/annual-and-interim-reports, accessed 12 September 2019). These genogroups not necessarily correlate with phylogenetic clustering of full-length HA trees as shown in Supplementary Figure S3. This phylogenetic tree presents 245 H1_pdm_N1_pdm_ sequences plus 15 reference sequences of human and porcine strains. It demonstrates numerous independent incursions of H1_pdm_N1_pdm_ into the pig population in Europe. Noticeable, only two of the many anthroponotic events achieved to establish persistent infection chains in pigs: one led to the emergence of the Papenburg/2010-like swH1N2 lineage [8,13,16] and the other to the Wachtum/2014-like swH1_pdm_N1_pdm_. Whereas virus isolates of sporadic, non-persistent spill-over infections show only single amino acid substitutions at the antigenic sites, the Papenburg/2010-like and the Wachtum/2014-like strains exhibit numerous amino acid exchanges (Supplementary Figure S5C). Amino acid substitutions of both virus groups correlate with altered antigenic properties. For the Papenburg/2010-like swH1N2, the lack of cross-reactivity with EA swH1N1 has been shown to be due to five substitutions, K159R, G172E, I183V, S202N and D204S [13,16], but on the basis of 79 strains of this cluster, additional substitutions have been identified, e.g., A90S, N142D/K/S, P154S, A158T/E, L178I, K180I, D185G, G187R, A203D, S207T, Q210K, A212T and R222K. All Papenburg/2010-like strains exhibit 5–10 substitutions in antigenic sites. The Wachtum/2014-like viruses show 11 amino acid substitutions in antigenic sites: P141T, N142D, K159S, G172T, N173D, S179N, K180I, D185N, S202A, S207R and D239N. In addition, both the Papenburg/2010-like and the Wachtum/2014-like viruses exhibit an additional potential glycosylation site at N179 and N202, respectively, which are located in antigenic sites Ca1 and Sb (Supplementary Figure S5C).

In order to investigate cross-reactivity of sporadic, anthroponotic H1_pdm_N1_pdm_ and Wachtum/2014-like swH1_pdm_N1_pdm_ viruses as well as EA swH1N1 and swH1_pdm_N2 viruses, immune sera and hyperimmune sera were established and tested in the HI assay. The data show different degrees in cross-reactivity but clearly indicate that the swH1_pdm_N2 as well as the Wachtum-2014-like swH1_pdm_N1_pdm_ viruses still exhibit only a very low cross-reactivity in hyperimmune sera to EA swH1N1 and human H1_pdm_N1_pdm_, whereas immune sera of Wachtum-2014-like swH1_pdm_N1_pdm_ viruses lack cross-reactivity to the other viruses (Table 1). The cross-reactive pattern between swH1_pdm_N2 and Wachtum-2014-like H1_pdm_N1_pdm_ is very low. Thus, two antigenic variant H1_pdm_Nx viruses circulate in German pigs.

The Schneiderkrug/2013-like swH1N1 are antigenically very similar to their parental Diepholz/2008-like swH1N2, i.e., they are characterized by P141S/K142N/T174I of antigenic site Sa (Supplementary Figure S5B).

Analysis of N-glycosylation patterns of HA proteins revealed three groups in the 1A lineage, which correspond to the sporadic anthroponotic H1_pdm_N1_pdm_, the Wachtum/2014-like viruses and the Papenburg/2010-like swH1N2 (Table 2). Furthermore, three patterns of 1B viruses are distinguishable: English swH1N2, Gent/1999-like swH1N2 and the Diepholz/2008-like swH1N2 together with the Schneiderkrug/2017-like swH1N1. Less conclusive is the distinction of the strains of lineage 1C. The viruses of sublineage 1C.2 and its sub-sublineages (1C.2.1, 1C.2.2, 1C.2.3) comprise one group but some variation is seen in up to four *N*-glycosylation sequons. Viruses of sublineage 1C.1 fall into two groups, the early isolates of 1979–1989, which are like avian H1N1, and the late strains isolated from 1991 onwards, which include virus isolates from the continent and the British Isles.

## 4. Discussion

**Figure 5:**
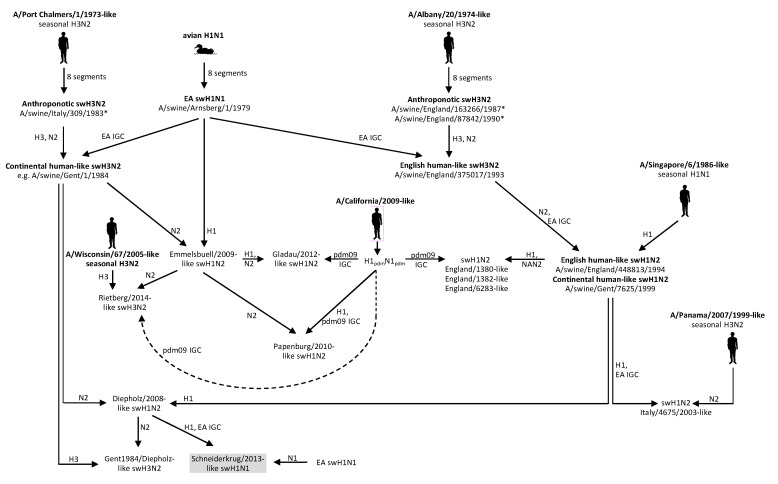
Evolution of German S-IAVs. Reassortment events leading to the main S-IAV lineages and the novel swH1N1 virus (presented in gray box) are shown. The Wachtum/2014-like swH1N1 are not shown here. Pictograms denote anthroponotic and spill-over infections. Asteriscs (*) indicate isolates with partial sequences.

Swine influenza is enzootic in Germany and many other European countries. A recent German 13-year long-term swine influenza surveillance conducted by the German FluResearchNet consortium revealed significant changes in the prevalence of circulating swH1N2 strains [8]. From 2008 onwards, several novel reassortants emerged in Germany, established stable infection chains and now co-circulate with pre-existing S-IAVs (Figure 5). In the present study, we focused on swH1N1 and made two remarkable observations: (1) a sustained circulation of swine-adapted H1_pdm_N1_pdm_, named Wachtum/2014-like swH1_pdm_N1_pdm_, which is a drift variant, and (2) emergence of a novel swH1N1 6 + 2 reassortant. This reassortant, designated Schneiderkrug/2013-like virus, is a derivative of the Diepholz/2008-like swH1N2 [8] and of an EA swH1N1 (donor of NA and NS). It is serologically inconspicuous, spread to three German States and co-circulates with the EA swH1N1 and the H1_pdm_N1_pdm_. This is interesting as the HA of these three viruses belong to three different genetic lineages, named 1A, 1B and 1C (compare Figure 2A), and hence, are antigenically heterologous. In addition, each of the three swH1N1 viruses co-circulates with swH1N2 viruses of the same HA sublineage. The respective co-circulating viruses are: (i) lineage 1 A: Wachtum/2014-like swH1_pdm_N1_pdm_ and Papenburg/2010-like swH1N2, (ii) lineage 1B: Schneiderkrug/2013-like swH1N1 and Diepholz/2008-like swH1N2 and (iii) lineage 1C: Emmelsbuell/2009-like plus Gladau/2012-like swH1N2 and the EA swH1N1. Different antigenic properties of the Wachtum/2014-like swH1_pdm_N1_pdm_ have been demonstrated in HI assays and correlate with specific substitutions of antigenic sites (Supplementary Figure S5C). Moreover, we observed clade-specific glycosylation patterns of the HA (Table 2) as evident from conserved/non-conserved NxS/T glycosylation sequons. Different N-glycosylation patterns may influence antigenic properties, binding to receptors, the HA/NA balance and transmissibility of the respective virus [33,34,35].

EA swH1N1 is prevalent in Germany and is known to cross-react at a certain level with H1_pdm_N1_pdm_. This cross-reactivity may have prevented a sustained circulation of H1_pdm_N1_pdm_ in pigs for several years, especially in pig herds that had contact with several EA swH1N1 viruses and therefore exhibited a broader cross-reactivity [15]. Sporadic anthropogenic infections of pigs with H1_pdm_N1_pdm_ occurred in Germany, mostly in areas with low-dense pig population, but no sustained circulation was observed. This changed with the upsurge of the Papenburg/2010-like swH1N2. Whereas the previous isolates of this reassortant exhibited five amino acid exchanges in their antigenic sites [13,16], the more recent strains have accumulated up to 10 amino acid substitutions (Supplementary Figure S5C). In 2014, the Wachtum/2014-like swH1_pdm_N1_pdm_ emerged with 11 amino acid substitutions in the antigenic sites plus an additional *N*-glycosylation site (Table 1). Whereas the Papenburg/2010-like viruses still display a very low degree of cross-reactivity to the human H1_pdm_N1_pdm_, the Wachtum/2014-like swH1_pdm_N1_pdm_ viruses neither cross-react with the EA swH1N1 nor with other H1_pdm_N1_pdm_ in immune sera. Hence, these viruses were the first H1_pdm_N1_pdm_ that accomplished a persistent infection chain in pigs. This feature may bear future problems in the pig industry, as no commercial vaccine is available to protect pigs sufficiently from this virus.

Notably, three mechanisms of S-IAV immune escape are observed: (i) The emergence of drift variants, which are not adequately matched by the available vaccines (e.g., the Wachtum/2014-like swH1_pdm_N1_pdm_), (ii) the emergence of reassortants and (iii) a combination of reassortment and antigenic drift (e.g., the Papenburg/2010-like swH1N2 [13]). The appearance of new S-IAV variants in quick succession make it increasingly difficult to ensure an all-encompassing vaccination of pigs. Beside novel swH1N1 variants, the diversity of the swH1N2 viruses has also increased in the last decade in Germany [8]. It is likely that changed swine farming practices and increased herd size as well as the invention of a trivalent swine influenza vaccine in Germany contributed to this diversity. Rose et al. and Ryt-Hansen et al. described increasing problems in herd management due to persistent S-IAV infection in both farrow-to-finish and farrow-to-nursery farms [29,30]. Only a few modeling studies addressed S-IAV dynamics in pig herds. Reynolds et al. investigated different vaccination strategies and indicated the failure to eliminate swine influenza if only partial protection is achieved by the vaccine due to circulating heterologous S-IAV strains [36]. Cador et al. demonstrated that the presence of maternally derived antibodies may lead to an extended duration of epidemics and batch-to-batch infection [37]. The problem is further aggravated by epidemiological models that had been calibrated against seroprevalence data from Dutch finishing pigs and pointed out that S-IAV may persist even in small pig herds [38]. The co-circulation of two distinct S-IAV strains was investigated in another study of Cador et al. [39]. Modeling different vaccination schemes and herd management strategies, these authors evaluated the probability of a co-infection event in France to circa 17%, and no vaccination strategy achieved S-IAV elimination within the farrow-to-finish pig herd.

These studies are compatible with our observations: repeated virus isolation within the same farm, isolation of different types and the emergence of reassortants are indicative of persistent infection of a herd despite vaccination. The unexpected occurrence of the Wachtum/2014-like drift variant with 11 substitutions in the antigenic sites of HA is concerning, as this indicates long-lasting inconspicuous circulation afore. The observed spread of this variant to neighboring German states suggests increasing enzootic circulation. In addition, such a drift variant bears a certain zoonotic risk, as the human IAV vaccine probably does not cover its antigenic properties. Our data once more indicate the necessity to intensify broader swine influenza surveillance.

## Figures and Tables

**Table 1:** Immunogenic pattern of Wachtum/2014-like swH1_pdm_N1_pdm_ in comparison to other H1N1 and H1N2 viruses detected in pigs in Germany (HI titer reciprocal).

	EA swH1N1	Sporadic, Anthropogenic H1_pdm_N1_pdm_	Papenburg/2010-Like H1_pdm_N2	Wachtum/2014-Like H1_pdm_N1_pdm_
**Immune Sera**				
EA swH1N1	197	<20	<20	<20
anthropogenic H1_pdm_N1_pdm_	21	106	34	<20
Papenburg/2010-like H1_pdm_N2	<20	49	485	<20
Wachtum/2014-like H1_pdm_N1_pdm_	<20	<20	<20	160
**Hyper Immune Sera**				
EA swH1N1	2560	80	160	80
anthropogenic H1_pdm_N1_pdm_	640	2560	640	320
Papenburg/2010-like H1_pdm_N2	80	320	2560	20
Wachtum/2014-like H1_pdm_N1_pdm_	160	40	20	2560

**Table 2:** *N*-Glycosylation pattern of H1 of European S-IAV.

	Amino Acid Position of Glycosylation Sequons (N-X-S/T)																	
	27–29	28–30	40–42	101–103	104–106	136–138	142–144 ^1^	172–174 ^1^	177–179 ^1^	179–181 ^2^	202–204 ^3^	212–214	286–288	291–293	293–295	304–306	498–500	557–559
**Lineage 1A**																		
anthroponotic H1_pdm_N1_pdm_	+	+	+	-	+	-	-	-	-	-	-	-	-	-	+	+	+	+
Wachtum/2014-like swH1_pdm_N1_pdm_	+	+	+	-	+	-	-	-	-	+	-	-	-	-	+	+	+	+
Papenburg/2010-like swH1N2	+	+	+	-	+	-	-	-	-	-	+	-	-	-	+	+	+	+
**Lineage 1B**																		
Schneiderkrug/2014-like swH1N1	+	+	+	-	-	-	+	-	+	-	-	-	+	-	-	+	+	+
Diepholz/2008-like swH1N2	+	+	+	-	-	-	+	-	+	-	-	-	+	-	-	+	+	+
Gent/1999-like swH1N2	+	+	+	-	-	-	-	+	+	-	-	-	+	-	-	+	+	+
England/448813/1994-like swH1N2	+	+	+	-	-	-	-	-	+	-	-	-	+	-	-	+	+	+
**Lineage 1C**																		
sublineage 1C.2 (EA swH1N1, swH1N2)	+	+	+	B	B	B	-	-	-	B	-	+	-	+	-	-	+	+
sublineage 1C.1, 1991–1998	+	+	+	-	+	-	-	-	-	B	-	-	-	+	-	-	+	+
sublineage 1C.1, 1979–1989	+	+	+	-	+	-	-	-	-	-	-	-	-	-	-	+	+	+
avian H1N1	+	+	+	-	+	-	-	-	-	-		-	-	-	-	+	+	+

^1^ part of antigenic site Sa, ^2^ part of antigenic site Ca1, ^3^ part of antigenic site Sb, B, both variants (with and without glycosylation sequons) are observed.

## Supplementary Materials

The following are available online at https://www.mdpi.com/1999-4915/12/7/762/s1, Supplementary Information S1: Legends to supplementary figures, Figure S1: Phylogenetic analysis and genotypes of 950 HAH1 sequences, Figure S2: Phylogenetic analysis of 577 NAN1 sequences, Figure S3: Phylogenetic analysis of 258 HAH1 sequences of lineage 1A, Figure S4: Detection of intratypic reassortment, Figure S5: Analysis of antigenic sites, Table S1: List of investigated strains.
