# Supplementary material for: Cocirculation of Swine H1N1 Influenza A Virus Lineages in Germany
Source: Viruses. 2020 Jul 15;12(7):762. doi: 10.3390/v12070762 (PMC7411773; doi:10.3390/v12070762)
Supplement: Supplementary file 1 [file viruses-12-00762-s001.zip › Zell_et_al_Supplementary_Files_revised/Fig S2.pdf]

Suppl. Fig. 2

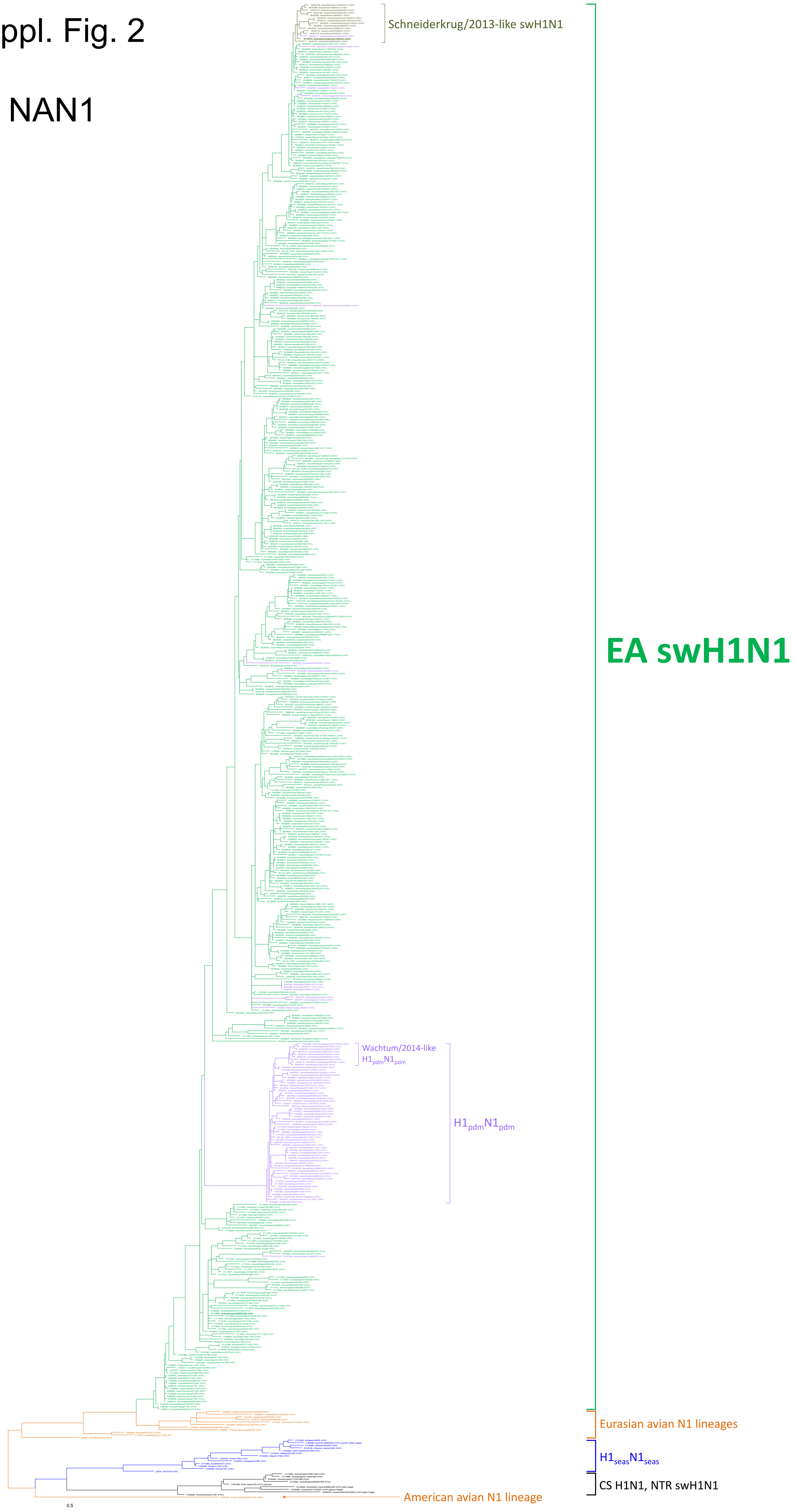
NAN1
Schneiderkrug/2013-like swH1N1
EA swH1N1
Wachtum/2014-like
H1pdmN1pdm
H1pdmN1pdm
Eurasian avian N1 lineages
H1seasN1seas
CS H1N1, NTR swH1N1
American avian N1 lineage
0.5
