# Supplementary material for: Cocirculation of Swine H1N1 Influenza A Virus Lineages in Germany
Source: Viruses. 2020 Jul 15;12(7):762. doi: 10.3390/v12070762 (PMC7411773; doi:10.3390/v12070762)

Suppl. Fig. 5C

lineage 1A

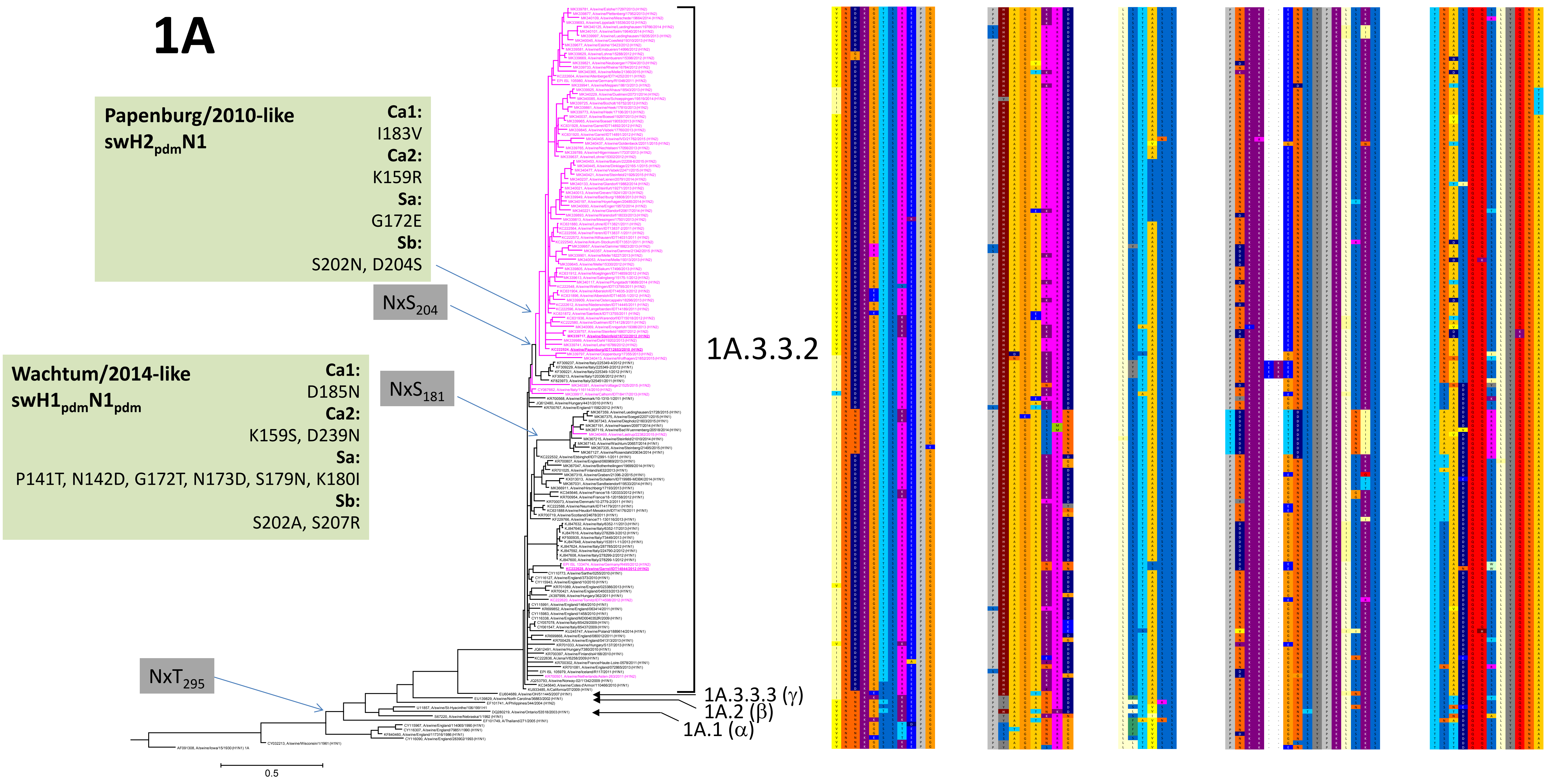

Supplement: Supplementary file 1 [file viruses-12-00762-s001.zip › Zell_et_al_Supplementary_Files_revised/Fig S5C.pdf]
