# Supplementary material for: Cocirculation of Swine H1N1 Influenza A Virus Lineages in Germany
Source: Viruses. 2020 Jul 15;12(7):762. doi: 10.3390/v12070762 (PMC7411773; doi:10.3390/v12070762)
Supplement: Supplementary file 1 [file viruses-12-00762-s001.zip › Zell_et_al_Supplementary_Files_revised/Legends to Supplementary Figures.pdf]

Cocirculation of swine H1N1 influenza A virus lineages in Germany

Roland Zell [1,*], Marco Groth [2], Andi Krumhbolz [1,3], Jeannette Lange [1,4], Anja Philipps [1,5] and Ralf Dürrwald [1,6]

[1] Section of Experimental Virology, Institute for Medical Microbiology, Jena University Hospital, Friedrich Schiller University Jena, D-07745 Jena, Germany; roland.zell@med.uni-jena.de

[2] CF DNA Sequencing, Leibniz Institute on Aging, Fritz Lipmann Institute, D-07745 Jena, Germany; marco.groth@leibniz-fli.de

[3] Present address: Institute of Infection Medicine, Kiel University and University Medical Center Schleswig-Holstein, D-24105 Kiel, Germany

[4] Present address: Paul-Ehrlich-Institut, D-63225 Langen, Germany

[5] Present address: Thermo Fisher Scientific GENEART GmbH, D-93059 Regensburg, Germany

[6] Present address: Robert Koch Institute, D-13353 Berlin, Germany

Legends to Supplementary Figures

Figure S1: Phylogenetic analysis and genotypes of 950 HAH1 sequences. 348 sequences generated by Jena SIVSI and 602 sequences of GenBank and EpiFlu databases were compiled. *Left panel:* Phylogenetic tree infered with MrBayes; substitution model: GTR+G+I; convergence was reached after 10.5 million generations. The tree presents strain designations and GenBank/EpiFlu accession numbers. Colour code of the tree: green, Eurasian avian swH1N1; light green, seasonal H1N1; olive, Schneiderkrug/2013-like swH1N1 strains; blue, English and continental swH1N2; light blue, Diepholz/2008-like swH1N2 strains; black, CS H1N1, North American triple reassortant swH1N1; purple, pandemic H1N1; magenta, pandemic swH1N2; ochre, avian H1. *Middle panel:* Genotypic analysis. Genotype designation according to Lu et al. (Nucl. Acids Res. 35:W275-W279, 2007). *Right panel:* Subtyping according to Anderson et al. (mSphere 1:e00275-16, 2016); major subgroups are also presented.

Figure S2: Phylogenetic analysis of 577 NAN1 sequences. 348 sequences generated by Jena SIVSI and 229sequences of GenBank and EpiFlu databases were compiled. The tree was infered with MrBayes; substitution model: GTR+g+I; convergence was reached after 2.5 million generations. The tree presents strain designations and GenBank/EpiFlu accession numbers. Color code: green, Eurasian avian swH1N1; ochre, avian N1 lineages; blue, seasonal H1N1; black, classical swine H1N1 and North American triple reassortant swH1N1; purple, pandemic H1N1 and reassortants with gene segments of pandemic H1N1; olive, Schneiderkrug/2013-like swH1N1.

Figure S3: Phylogenetic analysis of 258 HAH1 sequences of lineage 1A. 101 sequences generated by Jena SIVSI and 157 sequences of GenBank and EpiFlu databases were compiled. The tree was infered with MrBayes; substitution model GTR+g+I; convergence was reached after 15 million generations. The tree presents strain designations and GenBank/EpiFlu accession numbers. Color code: black, classical swine H1N1, North American triple reassortant H1N1, swine pandemic H1N1; green, human pandemic H1N1; magenta, swH1N2 with gene segments of pandemic H1N1. Subtyping according to Anderson et al. (mSphere 1:e00275-16, 2016) and the WHO nomenclature is presented to the right.

Figure S4: Detection of intratypic reassortment. Phylogenetic trees (identical to Suppl. Fig.s 1 and 2) of HAH1 (left) and NAN1 (right) are compared. Fifteen sequence clusters are indicated by different colours. Clusters #1, 2, 3, 6, 7, 11, 12, 13, 14, 15 are monophyletic, whereas clusters #4, 5, 8, 9, 10 are not.

Figure S5: Analysis of antigenic sites. **A.** Lineage 1C. **B.** Lineage 1B. **C.** Lineage 1A. In each figure, the **left panel** shows the phylogenetic tree as presented in Suppl. Fig. 1 but amended by indication of amino acid substitutions in the antigenic sites Ca1, Ca2, Cb, Sa, Sb as well as gain/loss of glycosylation sequons in the sequence context of antigenic sites (compare Table 2). Subtyping according to Anderson et al. (mSphere 1:e00275-16, 2016) is also presented. The right panel shows the antigenic sites of each virus strain; amino acids are colour-coded.
