# Supplementary material for: Cocirculation of Swine H1N1 Influenza A Virus Lineages in Germany
Source: Viruses. 2020 Jul 15;12(7):762. doi: 10.3390/v12070762 (PMC7411773; doi:10.3390/v12070762)
Supplement: Supplementary file 1 [file viruses-12-00762-s001.zip › Zell_et_al_Supplementary_Files_revised/Table_S1.pdf]

Supplementary Table 1: Virus strains, country, sampling dates and GenBank accession numbers

Strain		Sampling date	Seg. 1, Pol PB2	Seg. 2, Pol PB1	Seg. 3, Pol PA	Seg. 4, HA	Seg. 5, NP	Seg. 6, NA	Seg. 7, M	Seg. 8, NS
A/swine/Arnsberg/1/1979 (H1N1)	Germany	1979	KJ889297	KJ889298	KJ889299	KJ889300	KJ889301	KJ889302	KJ889303	KJ889304
A/swine/Arnsberg/6554/1979 (H1N1)	Germany	1979	KJ889305	KJ889306	KJ889307	KJ889308	KJ889309	KJ889310	KJ889311	KJ889312
A/swine/Netherlands/25/1980 (H1N1)	Netherlands	1980	KJ889321	KJ889322	KJ889323	KJ889324	KJ889325	KJ889326	KJ889327	KJ889328
A/swine/Marseille/2260/1980 (H1N1)	France	1980	KJ889313	KJ889314	KJ889315	KJ889316	KJ889317	KJ889318	KJ889319	KJ889320
A/swine/Arnsberg/3/1981 (H1N1)	Germany	1981	KJ889329	KJ889330	KJ889331	KJ889332	KJ889333	KJ889334	KJ889335	KJ889336
A/swine/Arnsberg/4/1981 (H1N1)	Germany	1981	KJ889337	KJ889338	KJ889339	KJ889340	KJ889341	KJ889342	KJ889343	KJ889344
A/swine/Arnsberg/7/1981 (H1N1)	Germany	1981	KJ889345	KJ889346	KJ889347	KJ889348	KJ889349	KJ889350	KJ889351	KJ889352
A/swine/Germany/S27/1981 (H1N1)	Germany	1981	KJ889361	KJ889362	KJ889363	KJ889364	KJ889365	KJ889366	KJ889367	KJ889368
A/swine/Potsdam/1/1981 (H1N1)	Germany	1981	KJ889376	KJ889377	KJ889378	KJ889379	KJ889380	KJ889381	EU478796	KJ889382
A/swine/Potsdam/15/1981 (H1N1)	Germany	1981	KJ889383	DQ836174	KJ889384	KJ889385	KJ889386	KJ889387	DQ186973	KJ889388
A/swine/Potsdam/268/1981 (H1N1)	Germany	1981	KJ889389	KJ889390	KJ889391	KJ889392	KJ889393	KJ889394	EU478797	KJ889395
A/swine/Hannover/1/1981 (H1N1)	Germany	1981	KJ889369	KJ889370	KJ889371	KJ889372	KJ889373	KJ889374	EU478795	KJ889375
A/swine/Italy/VI147/1981 (H1N1)	Italy	1981	KJ889396	KJ889397	KJ889398	KJ889399	KJ889400	KJ889401	KJ889402	KJ889403
A/swine/Germany/2/1981 (H1N1)	Germany	1981	KJ889353	KJ889354	KJ889355	KJ889356	KJ889357	KJ889358	KJ889359	KJ889360
A/swine/Italy/147/1981 (H1N1)	Italy	1981	KJ889404	KJ889405	KJ889406	KJ889407	KJ889408	KJ889409	KJ889410	KJ889411
A/swine/England/117316/1986 (H1N1)	United Kingdom	1986	KF840457	KF840458	KF840459	KF840460	KF840461	KF840462	KF840463	KF840464
A/swine/Schwerin/103/1989 (H1N1)	Germany	1989	MK364805	DQ836175	MK364806	MK364807	MK364808	MK364809	DQ186974	MK364810
A/swine/Gent/V230/1992 (H1N1)	Belgium	1992	MK364811	MK364812	MK364813	MK364814	MK364815	MK364816	EU487799	MK364817
A/swine/Bakum/5/1995 (H1N1)	Germany	1995	MK364818	DQ836176	MK364819	MK364820	MK364821	MK364822	DQ186975	MK364823
A/swine/Bakum/3543/1998 (H1N1)	Germany	1998	MK364824	MK364825	MK364826	MK364827	MK364828	MK364829	MK364830	MK364831
A/swine/Belzig/2/2001 (H1N1)	Germany	2001	MK364832	DQ836177	MK364833	MK364834	MK364835	MK364836	DQ186976	MK364837
A/swine/Haselünne/IDT2617-Pass1/2003 (H1N1)	Germany	09-Oct-2003	GQ161116	GQ161117	GQ161118	GQ161119	GQ161120	GQ161121	GQ161122	GQ161123
A/swine/Haselünne/IDT2617-Pass20/2003 (H1N1)	Germany	09-Oct-2003	GQ161109	GQ161110	GQ161111	GQ161112	GQ161113	GQ161114	EU478811	GQ161115
A/swine/Vechta/IDT2623/2003 (H1N1)	Germany	10-Oct-2003	MK364838	MK364839	MK364840	MK364841	MK364842	MK364843	EU478817	MK364844
A/swine/Geldern/IDT2888/2004 (H1N1)	Germany	06-Jan-2004	MK364845	MK364846	MK364847	MK364848	MK364849	MK364850	EU478821	MK364851
A/swine/Greven/IDT2889/2004 (H1N1)	Germany	05-Jan-2004	GQ161153	GQ161154	GQ161155	GQ161156	GQ161157	GQ161158	EU478823	GQ161159
A/swine/Gablingen/2998/2004 (H1N1)	Germany	26-Feb-2004	MK364852	MK364853	MK364854	MK364855	MK364856	MK364857	MK364858	MK364859
A/swine/Beienrode/3053/2004 (H1N1)	Germany	29-Mar-2004	MK364860	MK364861	MK364862	MK364863	MK364864	MK364865	MK364866	MK364867
A/swine/Kneheim/3066/2004 (H1N1)	Germany	05-Apr-2004	MK364868	MK364869	MK364870	MK364871	MK364872	MK364873	MK364874	MK364875
A/swine/Laer/IDT3893/2005 (H1N1)	Germany	15-Feb-2005	MK364876	MK364877	MK364878	MK364879	MK364880	MK364881	EU478837	MK364882
A/swine/Wohnste/IDT4093/2005 (H1N1)	Germany	27-Apr-2005	MK364883	MK364884	MK364885	MK364886	MK364887	MK364889	EU478845	MK364889
A/swine/Voglarn/IDT4096/2005 (H1N1)	Germany	09-May-2005	MK364890	MK364891	MK364892	MK364893	MK364894	MK364895	EU478844	MK364896
A/swine/Hemmingen/IDT4097/2005 (H1N1)	Germany	09-May-2005	MK364897	MK364898	MK364899	MK364900	MK364901	MK364902	EU478834	MK364903
A/swine/Kroge/IDT4192/2005 (H1N1)	Germany	17-Jun-2005	MK364904	MK364905	MK364906	MK364907	MK364908	MK364909	EU478836	MK364910
A/swine/Rheda-Wiedenbrück/4205/2005 (H1N1)	Germany	22-Jun-2005	MK364911	MK364912	MK364913	MK364914	MK364915	MK364916	MK364917	MK364918
A/swine/Passau/4207/2005 (H1N1)	Germany	22-Jun-2005	MK364919	MK364920	MK364921	MK364922	MK364923	MK364924	MK364925	MK364926
A/swine/Bad Rothenfelde/4255/2005 (H1N1)	Germany	05-Jul-2005	MK364927	MK364928	MK364929	MK364930	MK364931	MK364932	MK364933	MK364934
A/swine/Oldinghausen/4256/2005 (H1N1)	Germany	05-Jul-2005	MK364935	MK364936	MK364937	MK364938	MK364939	MK364940	MK364941	MK364942
A/swine/Nordhorn/4277/2005 (H1N1)	Germany	12-Jul-2005	MK364943	MK364944	MK364945	MK364946	MK364947	MK364948	MK364949	MK364950
A/swine/Zeitlarn/4287/2005 (H1N1)	Germany	13-Jul-2005	MK364951	MK364952	MK364953	MK364954	MK364955	MK364956	MK364957	MK364958
A/swine/Wenzendorf/4353/2005 (H1N1)	Germany	08-Aug-2005	MK364959	MK364960	MK364961	MK364962	MK364963	MK364964	MK364965	MK364966
A/swine/Großharie/4372/2005 H1N1)	Germany	16-Aug-2005	MK364967	MK364968	MK364969	MK364970	MK364971	MK364972	MK364973	MK364974
A/swine/Bocholt/4414/2005 (H1N1)	Germany	30-Aug-2005	MK364975	MK364976	MK364977	MK364978	MK364979	MK364980	MK364981	MK364982
A/swine/Osnabrück/4440/2005 (H1N1)	Germany	06-Sep-2005	MK364983	MK364984	MK364985	MK364986	MK364987	MK364988	MK364989	MK364990
A/swine/Haren/4509/2005 (H1N1)	Germany	28-Sep-2005	MK364991	MK364992	MK364993	MK364994	MK364995	MK364996	MK364997	MK364998
A/swine/Rotthalmünster/4564/2005 (H1N1)	Germany	14-Oct-2005	MK364999	MK365000	MK365001	MK365002	MK365003	MK365004	MK365005	MK365006
A/swine/Wenzendorf/4584/2005 (H1N1)	Germany	17-Oct-2005	MK365007	MK365008	MK365009	MK365010	MK365011	MK365012	MK365013	MK365014
A/swine/Visbek/4656/2005 (H1N1)	Germany	09-Nov-2005	MK365015	MK365016	MK365017	MK365018	MK365019	MK365020	MK365021	MK365022
A/swine/Krerensen/4662/2005 (H1N1)	Germany	14-Nov-2005	MK365023	MK365024	MK365025	MK365026	MK365027	MK365028	MK365029	MK365030
A/swine/Hemmoor/4674/2005 (H1N1)	Germany	21-Nov-2005	MK365031	MK365032	MK365033	MK365034	MK365035	MK365036	MK365037	MK365038
A/swine/Haselünne/4709/2005 (H1N1)	Germany	30-Nov-2005	MK365039	MK365040	MK365041	MK365042	MK365043	MK365044	MK365045	MK365046
A/swine/Engelsberg/4721/2005 (H1N1)	Germany	05-Dec-2005	MK365047	MK365048	MK365049	MK365050	MK365051	MK365052	MK365053	MK365054

A/swine/Laar/4772/2005 (H1N1)	Germany	14-Dec-2005	MK365055	MK365056	MK365057	MK365058	MK365059	MK365060	MK365061	MK365062
A/swine/Brakel/4806/2006 (H1N1)	Germany	23-Dec-2005	MK365063	MK365064	MK365065	MK365066	MK365067	MK365068	MK365069	MK365070
A/swine/Günne/4847/2006 (H1N1)	Germany	12-Jan-2006	MK365071	MK365072	MK365073	MK365074	MK365075	MK365076	MK365077	MK365078
A/swine/Ense/4848/2006 (H1N1)	Germany	12-Jan-2006	MK365079	MK365080	MK365081	MK365082	MK365083	MK365084	MK365085	MK365086
A/swine/Meppen/4919/2006 (H1N1)	Germany	31-Jan-2006	MK365087	MK365088	MK365089	MK365090	MK365091	MK365092	MK365093	MK365094
A/swine/Melle/5003/2006 (H1N1)	Germany	20-Feb-2006	MK365095	MK365096	MK365097	MK365098	MK365099	MK365100	MK365101	MK365102
A/swine/Lichtenau/5045/2006 (H1N1)	Germany	07-Mar-2006	MK365103	MK365104	MK365105	MK365106	MK365107	MK365108	MK365109	MK365110
A/swine/Nordkirchen /5062/2006 (H1N1)	Germany	10-Mar-2006	MK365111	MK365112	MK365113	MK365114	MK365115	MK365116	MK365117	MK365118
A/swine/Holzheim/5102/2006 (H1N1)	Germany	22-Mar-2006	MK365119	MK365120	MK365121	MK365122	MK365123	MK365124	MK365125	MK365126
A/swine/Steinhorst/5129/2006 (H1N1)	Germany	04-Apr-2006	MK365127	MK365128	MK365129	MK365130	MK360131	MK365132	MK365133	MK365134
A/swine/Ortensburg/5144/2006 (H1N1)	Germany	10-Apr-2006	MK365135	MK365136	MK365137	MK365138	MK365139	MK365140	MK365141	MK365142
A/swine/Südlohn/5508/2006 (H1N1)	Germany	30-Aug-2006	MK365143	MK365144	MK365145	MK365146	MK365147	MK365148	MK365149	MK365150
A/swine/Bocholt/5533/2006 (H1N1)	Germany	05-Sep-2006	MK365151	MK365152	MK365153	MK365154	MK365155	MK365156	MK365157	MK365158
A/swine/Ramsberg/5544/2006 (H1N1)	Germany	11-Sep-2006	MK365159	MK365160	MK365161	MK365162	MK365163	MK365164	MK365165	MK365166
A/swine/Friesoythe/5548/2006 (H1N1)	Germany	11-Sep-2006	MK365167	MK365168	MK365169	MK365170	MK365171	MK365172	MK365173	MK365174
A/swine/Friesoythe/5549/2006 (H1N1)	Germany	11-Sep-2006	MK365175	MK365176	MK365177	MK365178	MK365179	MK365180	MK365181	MK365182
A/swine/Kassel/5550/2006 (H1N1)	Germany	13-Sep-2006	MK365183	MK365184	MK365185	MK365186	MK365187	MK365188	MK365189	MK365190
A/swine/Altenaffeln/5551/2006 (H1N1)	Germany	15-Sep-2006	MK365191	MK365192	MK365193	MK365194	MK365195	MK365196	MK365197	MK365198
A/swine/Ostercappeln/5553/2006 (H1N1)	Germany	20-Sep-2006	MK365199	MK365200	MK365201	MK365202	MK365203	MK365204	MK365205	MK365206
A/swine/Balve/5603/2006 (H1N1)	Germany	16-Oct-2006	MK365207	MK365208	MK365209	MK365210	MK365211	MK365212	MK365213	MK365214
A/swine/Bad Griesbach/IDT5604/2006 (H1N1)	Germany	17-Oct-2006	MK365215	MK365216	MK365217	MK365218	MK365219	MK365220	EU478846	MK365221
A/swine/Gütersloh/5610/2006(H1N1)	Germany	17-Oct-2006	MK365222	MK365223	MK365224	MK365225	MK365226	MK365227	MK365228	MK365229
A/swine/Rotthalmünster/5616/2006 (H1N1)	Germany	23-Oct-2006	MK365230	MK365231	MK365232	MK365233	MK365234	MK365235	MK365236	MK365237
A/swine/Feldbach/5625/2006 (H1N1)	Austria	22-Oct-2006	MK365238	MK365239	MK365240	MK365241	MK365242	MK365243	MK365244	MK365245
A/swine/Schloß Holte/5647/2006 (H1N1)	Germany	06-Nov-2006	MK365246	MK365247	MK365248	MK365249	MK365250	MK365251	MK365252	MK365253
A/swine/Nordhorn/5657/2006 (H1N1)	Germany	03-Nov-2006	MK365254	MK365255	MK365256	MK365257	MK365258	MK365259	MK365260	MK365261
A/swine/Gottfrieding/5732/2006 (H1N1)	Germany	26-Nov-2006	MK365262	MK365263	MK365264	MK365265	MK365266	MK365267	MK365268	MK365269
A/swine/Borgholzhausen/5751/2006 (H1N1)	Germany	04-Dec-2006	MK365270	MK365271	MK365272	MK365273	MK365274	MK365275	MK365276	MK365277
A/swine/Wadersloh/5761/2006 (H1N1)	Germany	06-Dec-2006	MK365278	MK365279	MK365280	MK365281	MK365282	MK365283	MK365284	MK365285
A/swine/Ennigerloh/IDT5803/2006 (H1N1)	Germany	13-Dec-2006	MK365286	MK365287	MK365288	GQ161139	MK365289	GQ161140	MK365290	MK365291
A/swine/Geseke/6004/2007 (H1N1)	Germany	15-Mar-2007	MK365292	MK365293	MK365294	MK365295	MK365296	MK365297	MK365298	MK365299
A/swine/Bad Wünnenberg/6005/2007 (H1N1)	Germany	19-Mar-2007	MK365300	MK365301	MK365302	MK365303	MK365304	MK365305	MK365306	MK365307
A/swine/Bielefeld/6033/2007 (H1N1)	Germany	26-Mar-2007	MK365308	MK365309	MK365310	MK365311	MK365312	MK365313	MK365314	MK365315
A/swine/Schmallenberg/6049/2007 (H1N1)	Germany	03-Apr-2007	MK365316	MK365317	MK365318	MK365319	MK365320	MK365321	MK365322	MK365323
A/swine/Fronhausen/6059/2007 (H1N1)	Germany	12-Apr-2007	MK365324	MK365325	MK365326	MK365327	MK365328	MK365329	MK365330	MK365331
A/swine/Feldbach/6072/2007 (H1N1)	Austria	15-Apr-2007	MK365332	MK365333	MK365334	MK365335	MK365336	MK365337	MK365338	MK365339
A/swine/Wülknitz/6141/2007 (H1N1)	Germany	11-May-2007	MK365340	MK365341	MK365342	MK365343	MK365344	MK365345	MK365346	MK365347
A/swine/Nordkirchen/6167/2007 (H1N1)	Germany	16-May-2007	MK365348	MK365349	MK365350	MK365351	MK365352	MK365353	MK365354	MK365355
A/swine/Lippetal/6292/2007 (H1N1)	Germany	17-Jul-2007	MK365356	MK365357	MK365358	MK365359	MK365360	MK365361	MK365362	MK365363
A/swine/Marsberg/6307/2007 (H1N1)	Germany	24-Jul-2007	MK365364	MK365365	MK365366	MK365367	MK365368	MK365369	MK365370	MK365371
A/swine/Schapen/6332/2007 (H1N1)	Germany	08-Aug-2007	MK365372	MK365373	MK365374	MK365375	MK365376	MK365377	MK365378	MK365379
A/swine/Borgholzhausen/6371/2007 (H1N1)	Germany	17-Aug-2007	MK365380	MK365381	MK365382	MK365383	MK365384	MK365385	MK365386	MK365387
A/swine/Paderborn/6373/2007 (H1N1)	Germany	21-Aug-2007	MK365388	MK365389	MK365390	MK365391	MK365392	MK365393	MK365394	MK365395
A/swine/Daseburg/6531/2007 (H1N1)	Germany	09-Oct-2007	MK365396	MK365397	MK365398	MK365399	MK365400	MK365401	MK365402	MK365403
A/swine/Bösel/6543/2007 (H1N1)	Germany	11-Oct-2007	MK365404	MK365405	MK365406	MK365407	MK365408	MK365409	MK365410	MK365411
A/swine/Bad Rothenfelde/6622/2007 (H1N1)	Germany	05-Nov-2007	MK365412	MK365413	MK365414	MK365415	MK365416	MK365417	MK365418	MK365419
A/swine/Sundern/6655a/2007 (H1N1)	Germany	22-Nov-2007	MK365420	MK365421	MK365422	MK365423	MK365424	MK365425	MK365426	MK365427
A/swine/Brakel/6672/2007 (H1N1)	Germany	27-Nov-2007	MK365428	MK365429	MK365430	MK365431	MK365432	MK365433	MK365434	MK365435
A/swine/Alsfeld/6673/2007 (H1N1)	Germany	28-Nov-2007	MK365436	MK365437	MK365438	MK365439	MK365440	MK365441	MK365442	MK365443
A/swine/Everswinkel/6774/2008 (H1N1)	Germany	15-Jan-2008	MK365444	MK365445	MK365446	MK365447	MK365448	MK365449	MK365450	MK365451
A/swine/Minden-Lübbecke/6810/2008 (H1N1)	Germany	28-Jan-2008	MK365452	MK365453	MK365454	MK365455	MK365456	MK365457	MK365458	MK365459
A/swine/Steinau/6818/2008 (H1N1)	Germany	31-Jan-2008	MK365460	MK365461	MK365462	MK365463	MK365464	MK365465	MK365466	MK365467
A/swine/Emtinghausen/6821/2008 (H1N1)	Germany	28-Jan-2008	MK365468	MK365469	MK365470	MK365471	MK365472	MK365473	MK365474	MK365475
A/swine/Hornstorf/6849/2008 (H1N1)	Germany	12-Feb-2008	MK365476	MK365477	MK365478	MK365479	MK365480	MK365481	MK365482	MK365483
A/swine/Soest/6948/2008 (H1N1)	Germany	12-Mar-2008	MK365484	MK365485	MK365486	MK365487	MK365488	MK365489	MK365490	MK365491
A/swine/Vreden/6986/2008 (H1N1)	Germany	31-Mar-2008	MK365492	MK365493	MK365494	MK365495	MK365496	MK365497	MK365498	MK365499

A/swine/Twistringen/6991/2008 (H1N1)	Germany	03-Apr-2008	MK365500	MK365501	MK365502	MK365503	MK365504	MK365505	MK365506	MK365507
A/swine/Twistringen/7029/2008 (H1N1)	Germany	15-Apr-2008	MK365508	MK365509	MK365510	MK365511	MK365512	MK365513	MK365514	MK365515
A/swine/Homberg/7039/2008 (H1N1)	Germany	22-Apr-2008	MK365516	MK365517	MK365518	MK365519	MK365520	MK365521	MK365522	MK365523
A/swine/Twistringen/7044/2008 (H1N1)	Germany	22-Apr-2008	MK365524	MK365525	MK365526	MK365527	MK365528	MK365529	MK365530	MK365531
A/swine/Heiden/7064/2008 (H1N1)	Germany	30-Apr-2008	MK365532	MK365533	MK365534	MK365535	MK365536	MK365537	MK365538	MK365539
A/swine/Uelzen/7066/2008 (H1N1)	Germany	25-Apr-2008	MK365540	MK365541	MK365542	MK365543	MK365544	MK365545	MK365546	MK365547
A/swine/Klosterseelte/7129/2008 (H1N1)	Germany	29-May-2008	MK365548	MK365549	MK365550	MK365551	MK365552	MK356553	MK365554	MK365555
A/swine/Dinklage/7240/2008 (H1N1)	Germany	09-Jul-2008	MK365556	MK365557	MK365558	MK365559	MK365560	MK365561	MK365562	MK365563
A/swine/Brakel/7488a/2008 (H1N1)	Germany	02-Sep-2008	MK365564	MK365565	MK365566	MK365567	MK365568	MK365569	MK365570	MK365571
A/swine/Horneburg/7489/2008 (H1N1)	Germany	01-Sep-2008	MK365572	MK365573	MK365574	MK365575	MK365576	MK365577	MK365578	MK365579
A/swine/Harsefeld/7490/2008 (H1N1)	Germany	27-Aug-2008	MK365580	MK365581	MK365582	MK365583	MK365584	MK365585	MK365586	MK365587
A/swine/Malching/7497/2008 (H1N1)	Germany	03-Sep-2008	MK365588	MK365589	MK365590	MK365591	MK365592	MK365593	MK365594	MK365595
A/swine/Pocking/7498/2008 (H1N1)	Germany	03-Sep-2008	MK365596	MK365597	MK365598	MK365599	MK365600	MK365601	MK365602	MK365603
A/swine/Meppen/7555/2008 (H1N1)	Germany	15-Sep-2008	MK365604	MK365605	MK365606	MK365607	MK365608	MK365609	MK365610	MK365611
A/swine/Melle/7590/2008 (H1N1)	Germany	24-Sep-2008	MK365612	MK365613	MK365614	MK365615	MK365616	MK365617	MK365618	MK365619
A/swine/Spenge/7616/2008 (H1N1)	Germany	30-Sep-2008	MK365620	MK365621	MK365622	MK365623	MK365624	MK365625	MK365626	MK365627
A/swine/Dötlingen/7655/2008 (H1N1)	Germany	06-Oct-2008	MK365628	MK365629	MK365630	MK365631	MK365632	MK365633	MK365634	MK365635
A/swine/Diemelstadt/7814/2008 (H1N1)	Germany	24-Oct-2008	MK365636	MK365637	MK365638	MK365639	MK365640	MK365641	MK365642	MK365643
A/swine/Melle/7831/2008 (H1N1)	Germany	27-Oct-2008	MK365644	MK365645	MK365646	MK365647	MK365648	MK365649	MK365650	MK365651
A/swine/Wetschen/7847/2008 (H1N1)	Germany	27-Oct-2008	MK365652	MK365653	MK365654	MK365655	MK365656	MK365657	MK365658	MK365659
A/swine/Emstek/8155/2008 (H1N1)	Germany	01-Dec-2008	MK365660	MK365661	MK365662	MK365663	MK365664	MK365665	MK365666	MK365667
A/swine/Freren/8297a/2008 (H1N1)	Germany	10-Dec-2008	MK365668	MK365669	MK365670	MK365671	MK365672	MK365673	MK365674	MK365675
A/swine/Poland/Y1/2008 (H1N1)	Poland	2008	MK365676	MK365677	MK365678	MK365679	MK365680	MK365681	MK365682	MK365683
A/swine/Wittmund/8498/2009 (H1N1)	Germany	12-Jan-2009	MK365684	MK365685	MK365686	MK365687	MK365688	MK365689	MK365690	MK365691
A/swine/Feldbach/8647/2009 (H1N1)	Austria	22-Jan-2009	MK365692	MK365693	MK365694	MK365695	MK365696	MK365697	MK365698	MK365699
A/swine/Berge/8685/2009 (H1N1)	Germany	27-Jan-2009	MK365700	MK365701	MK365702	MK365703	MK365704	MK365705	MK365706	MK365707
A/swine/Emtinghausen/8705/2009 (H1N1)	Germany	28-Jan-2009	MK365708	MK365709	MK365710	MK365711	MK365712	MK365713	MK365714	MK365715
A/swine/Gütersloh/8724a/2009 (H1N1)	Germany	03-Feb-2009	MK365716	MK365717	MK365718	MK365719	MK365720	MK365721	MK365722	MK365723
A/swine/Heinsberg/8905/2009 (H1N1)	Germany	18-Feb-2009	MK365724	MK365725	MK365726	MK365727	MK365728	MK365729	MK365730	MK365731
A/swine/Cloppenburg/8967a/2009 (H1N1)	Germany	23-Feb-2009	MK365732	MK365733	MK365734	MK365735	MK365736	MK365737	MK365738	MK365739
A/swine/Bartenshagen/9002/2009 (H1N1)	Germany	26-Feb-2009	MK365740	MK365741	MK365742	MK365743	MK365744	MK365745	MK365746	MK365747
A/swine/Enger/9149/2009 (H1N1)	Germany	11-Mar-2009	MK365748	MK365749	MK365750	MK365751	MK365752	MK365753	MK365754	MK365755
A/swine/Neudörpen/9185/2009 (H1N1)	Germany	16-Mar-2009	MK365756	MK365757	MK365758	MK365759	MK365760	MK365761	MK365762	MK365763
A/swine/Niebüll/9201-1/2009 (H1N1)	Germany	19-Mar-2009	MK365764	MK365765	MK365766	MK365767	MK365768	MK365769	MK365770	MK365771
A/swine/Stadtlohn/9247/2009 (H1N1)	Germany	20-Mar-2009	MK365772	MK365773	MK365774	MK365775	MK365776	MK365777	MK365778	MK365779
A/swine/Rheine/9278/2009 (H1N1)	Germany	27-Mar-2009	MK365780	MK365781	MK365782	MK365783	MK365784	MK365785	MK365786	MK365787
A/swine/Engelsberg/9321/2009 (H1N1)	Germany	27-Mar-2009	MK365788	MK365789	MK365790	MK365791	MK365792	MK365793	MK365794	MK365795
A/swine/Herford/9359/2009 (H1N1)	Germany	06-Apr-2009	MK365796	MK365797	MK365798	MK365799	MK365800	MK365801	MK365802	MK365803
A/swine/Osnabrueck/9368/2009 (H1N1)	Germany	07-Apr-2009	MK365804	MK365805	MK365806	MK365807	MK365808	MK365809	MK365810	MK365811
A/swine/Goch/9369/2009 (H1N1)	Germany	08-Apr-2009	MK365812	MK365813	MK365814	MK365815	MK365816	MK365817	MK365818	MK365819
A/swine/Warendorf/9387/2009 (H1N1)	Germany	09-Apr-2009	MK365820	MK365821	MK365822	MK365823	MK365824	MK365825	MK365826	MK365827
A/swine/Frontenhausen/9388/2009 (H1N1)	Germany	02-Apr-2009	MK365828	MK365829	MK365830	MK365831	MK365832	MK365833	MK365834	MK365835
A/swine/Freren/9658/2009 (H1N1)	Germany	26-May-2009	MK365836	MK365837	MK365838	MK365839	MK365840	MK365841	MK365842	MK365843
A/swine/Egglham/9790/2009 (H1N1)	Germany	21-Jun-2009	MK365844	MK365845	MK365846	MK365847	MK365848	MK365849	MK365850	MK365851
A/swine/Dornburg/10061/2009 (H1N1)	Germany	05-Aug-2009	MK365852	MK365853	MK365854	MK365855	MK365856	MK365857	MK365858	MK365859
A/swine/Warendorf/10105/2009 (H1N1)	Germany	11-Aug-2009	MK365860	MK365861	MK365862	MK365863	MK365864	MK365865	MK365866	MK365867
A/swine/Meppen/10115/2009 (H1N1)	Germany	10-Aug-2009	MK365868	MK365869	MK365870	MK365871	MK365872	MK365873	MK365874	MK365875
A/swine/Ennigerloh/10277/2009 (H1N1)	Germany	02-Sep-2009	MK365876	MK365877	MK365878	MK365879	MK365880	MK365881	MK365882	MK365883
A/swine/Egersheim/10464/2009 (H1N1)	Germany	16-Sep-2009	MK365884	MK365885	MK365886	MK365887	MK365888	MK365889	MK365890	MK365891
A/swine/Lohne/10524/2009 (H1N1)	Germany	29-Sep-2009	MK365892	MK365893	MK365894	MK365895	MK365896	MK365897	MK365898	MK365899
A/swine/Sögel/10604/2009 (H1N1)	Germany	05-Oct-2009	MK365900	MK365901	MK365902	MK365903	MK365904	MK365905	MK365906	MK365907
A/swine/Harsefeld/10671/2009 (H1N1)	Germany	16-Oct-2009	MK365908	MK365909	MK365910	MK365911	MK365912	MK365913	MK365914	MK365915
A/swine/Brakel/10686/2009 (H1N1)	Germany	13-Oct-2009	MK365916	MK365917	MK365918	MK365919	MK365920	MK365921	MK365922	MK365923
A/swine/Horneburg/10748/2009 (H1N1)	Germany	26-Oct-2009	MK365924	MK365925	MK365926	MK365927	MK365928	MK365929	MK365930	MK365931
A/swine/Borken/10752a/2009 (H1N1)	Germany	26-Oct-2009	MK365932	MK365933	MK365934	MK365935	MK365936	MK365937	MK365938	MK365939
A/swine/Lohne/10840/2009 (H1N1)	Germany	03-Nov-2009	MK365940	MK365941	MK365942	MK365943	MK365944	MK365945	MK365946	MK365947

A/swine/Norden/11211/2009 (H1N1)	Germany	11-Dec-2009	MK365948	MK365949	MK365950	MK365951	MK365952	MK365953	MK365954	MK365955
A/swine/Iserlohn/11287/2010 (H1N1)	Germany	11-Jan-2010	MK365956	MK365957	MK365958	MK365959	MK365960	MK365961	MK365962	MK365963
A/swine/Lohne/11566/2010 (H1N1)	Germany	24-Feb-2010	MK365964	MK365965	MK365966	MK365967	MK365968	MK365969	MK365970	MK365971
A/swine/Molbergen/11837a/2010 (H1N1)	Germany	15-Apr-2010	MK365972	MK365973	MK365974	MK365975	MK365976	MK365977	MK365978	MK365979
A/swine/Lohne/11843/2010 (H1N1)	Germany	16-Apr-2010	MK365980	MK365981	MK365982	MK365983	MK365984	MK365985	MK365986	MK365987
A/swine/Lohne/11867/2010 (H1N1)	Germany	20-Apr-2010	MK365988	MK365989	MK365990	MK365991	MK365992	MK365993	MK365994	MK365995
A/swine/Visbek/11951-1/2010 (H1N1)	Germany	14-May-2010	MK365996	MK365997	MK365998	MK365999	MK366000	MK366001	MK366002	MK366003
A/swine/Salzkotten/12021/2010 (H1N1)	Germany	01-Jun-2010	MK366004	MK366005	MK366006	MK366007	MK366008	MK366009	MK366010	MK366011
A/swine/Molbergen/12127/2010 (H1N1)	Germany	25-Jun-2010	MK366012	MK366013	MK366014	MK366015	MK366016	MK366017	MK366018	MK366019
A/swine/Raesefeld/12200-1/2010 (H1N1)	Germany	23-Jul-2010	MK366020	MK366021	MK366022	MK366023	MK366024	MK366025	MK366026	MK366027
A/swine/Raesefeld/12200-2/2010 (H1N1)	Germany	23-Jul-2010	MK366028	MK366029	MK366030	MK366031	MK366032	MK366033	MK366034	MK366035
A/swine/Brilon/12263-1/2010 (H1N1)	Germany	05-Aug-2010	MK366036	MK366037	MK366038	MK366039	MK366040	MK366041	MK366042	MK366043
A/swine/Brilon/12263-2/2010 (H1N1)	Germany	05-Aug-2010	MK366044	MK366045	MK366046	MK366047	MK366048	MK366049	MK366050	MK366051
A/swine/Gnas/12273/2010 (H1N1)	Austria	09-Aug-2010	MK366052	MK366053	MK366054	MK366055	MK366056	MK366057	MK366058	MK366059
A/swine/Elsten/12291/2010 (H1N1)	Germany	13-Aug-2010	MK366060	MK366061	MK366062	MK366063	MK366064	MK366065	MK366066	MK366067
A/swine/Lohne/12330/2010 (H1N1)	Germany	23-Aug-2010	MK366068	MK366069	MK366070	MK366071	MK366072	MK366073	MK366074	MK366075
A/swine/Vechta/12368/2010 (H1N1)	Germany	01-Sep-2010	MK366076	MK366077	MK366078	MK366079	MK366080	MK366081	MK366082	MK366083
A/swine/Haren/12382-1/2010 (H1N1)	Germany	08-Sep-2010	MK366084	MK366085	MK366086	MK366087	MK366088	MK366089	MK366090	MK366091
A/swine/Haren/12382-2/2010 (H1N1)	Germany	08-Sep-2010	MK366092	MK366093	MK366094	MK366095	MK366096	MK366097	MK366098	MK366099
A/swine/Friesoythe/12416/2010 (H1N1)	Germany	13-Sep-2010	MK366100	MK366101	MK366102	MK366103	MK366104	MK366105	MK366106	MK366107
A/swine/Altenberge/12445-1/2010 (H1N1)	Germany	17-Sep-2010	MK366108	MK366109	MK366110	MK366111	MK366112	MK366113	MK366114	MK366115
A/swine/Altenberge/12445-2/2010 (H1N1)	Germany	17-Sep-2010	MK366116	MK366117	MK366118	MK366119	MK366120	MK366121	MK366122	MK366123
A/swine/Höxter/12469/2010 (H1N1)	Germany	24-Sep-2010	MK366124	MK366125	MK366126	MK366127	MK366128	MK366129	MK366130	MK366131
A/swine/Aulendorf/12502/2010 (H1N1)	Germany	04-Oct-2010	MK366132	MK366133	MK366134	MK366135	MK366136	MK366137	MK366138	MK366139
A/swine/Warendorf/12524/2010 (H1N1)	Germany	08-Oct-2010	MK366140	MK366141	MK366142	MK366143	MK366144	MK366145	MK366146	MK366147
A/swine/Raddestorf/12564/2010 (H1N1)	Germany	13-Oct-2010	MK366148	MK366149	MK366150	MK366151	MK366152	MK366153	MK366154	MK366155
A/swine/Borchen/12595a/2010 (H1N1)	Germany	21-Oct-2010	MK366156	MK366157	MK366158	MK366159	MK366160	MK366161	MK366162	MK366163
A/swine/Borken/12666-1/2010 (H1N1)	Germany	04-Nov-2010	MK366164	MK366165	MK366166	MK366167	MK366168	MK366169	MK366170	MK366171
A/swine/Borken/12666-2/2010 (H1N1)	Germany	04-Nov-2010	MK366172	MK366173	MK366174	MK366175	MK366176	MK366177	MK366178	MK366179
A/swine/Balge/12670/2010 (H1N1)	Germany	09-Nov-2010	MK366180	MK366181	MK366182	MK366183	MK366184	MK366185	MK366186	MK366187
A/swine/Rahden/12686/2010 (H1N1)	Germany	10-Nov-2010	MK366188	MK366189	MK366190	MK366191	MK366192	MK366193	MK366194	MK366195
A/swine/Haren/12687/2010 (H1N1)	Germany	11-Nov-2010	MK366196	MK366197	MK366198	MK366199	MK366200	MK366201	MK366202	MK366203
A/swine/Altenberge/12724/2010 (H1N1)	Germany	22-Nov-2010	MK366204	MK366205	MK366206	MK366207	MK366208	MK366209	MK366210	MK366211
A/swine/Geeste/12729/2010 (H1N1)	Germany	22-Nov-2010	MK366212	MK366213	MK366214	MK366215	MK366216	MK366217	MK366218	MK366219
A/swine/Herzlake/12861/2011 (H1N1)	Germany	03-Jan-2011	MK366220	MK366221	MK366222	MK366223	MK366224	MK366225	MK366226	MK366227
A/swine/Ostbevern/12867-1/2011 (H1N1)	Germany	04-Jan-2011	MK366228	MK366229	MK366230	MK366231	MK366232	MK366233	MK366234	MK366235
A/swine/Ostbevern/12867-2/2011 (H1N1)	Germany	04-Jan-2011	MK366236	MK366237	MK366238	MK366239	MK366240	MK366241	MK366242	MK366243
A/swine/Bösel/12937/2011 (H1N1)	Germany	25-Jan-2011	MK366244	MK366245	MK366246	MK366247	MK366248	MK366249	MK366250	MK366251
A/swine/Ebbinghof/IDT12991-1/2011 (H1N1)	Germany	07-Feb-2011	KC222529	KC222530	KC222531	KC222532	KC222533	KC222534	KC222535	KC222536
A/swine/Goldenstedt/13046/2011 (H1N1)	Germany	18-Feb-2011	MK366252	MK366253	MK366254	MK366255	MK366256	MK366257	MK366258	MK366259
A/swine/Nordhorn/13095-1/2011 (H1N1)	Germany	01-Mar-2011	MK366260	MK366261	MK366262	MK366263	MK366264	MK366265	MK366266	MK366267
A/swine/Geseke/13124/2011 (H1N1)	Germany	01-Mar-2011	MK366268	MK366269	MK366270	MK366271	MK366272	MK366273	MK366274	MK366275
A/swine/Barnstorf/13130/2011 (H1N1)	Germany	09-Mar-2011	MK366276	MK366277	MK366278	MK366279	MK366280	MK366281	MK366282	MK366283
A/swine/Geseke/13216/2011 (H1N1)	Germany	29-Mar-2011	MK366284	MK366285	MK366286	MK366287	MK366288	MK366289	MK366290	MK366291
A/swine/Norden/13225-1/2011 (H1N1)	Germany	01-Apr-2011	MK366292	MK366293	MK366294	MK366295	MK366296	MK366297	MK366298	MK366299
A/swine/Norden/13236-2/2011 (H1N1)	Germany	04-Apr-2011	MK366300	MK366301	MK366302	MK366303	MK366304	MK366305	MK366306	MK366307
A/swine/Warstein/13455/2011 (H1N1)	Germany	24-May-2011	MK366308	MK366309	MK366310	MK366311	MK366312	MK366313	MK366314	MK366315
A/swine/Bakum/13456/2011 (H1N1)	Germany	24-May-2011	MK366316	MK366317	MK366318	MK366319	MK366320	MK366321	MK366322	MK366323
A/swine/Lohne/13474/2011 (H1N1)	Germany	26-May-2011	MK366324	MK366325	MK366326	MK366327	MK366328	MK366329	MK366330	MK366331
A/swine/Wasserleben/13484/2011 (H1N1)	Germany	30-May-2011	MK366332	MK366333	MK366334	MK366335	MK366336	MK366337	MK366338	MK366339
A/swine/Ascheberg/13532/2011 (H1N1)	Germany	16-Jun-2011	MK366340	MK366341	MK366342	MK366343	MK366344	MK366345	MK366346	MK366347
A/swine/Erwitte/13614/2011 (H1N1)	Germany	04-Jul-2011	MK366348	MK366349	MK366350	MK366351	MK366352	MK366353	MK366354	MK366355
A/swine/Lohne/13624/2011 (H1N1)	Germany	15-Jul-2011	MK366356	MK366357	MK366358	MK366359	MK366360	MK366361	MK366362	MK366363
A/swine/Norden/13638/2011 (H1N1)	Germany	19-Jul-2011	MK366364	MK366365	MK366366	MK366367	MK366368	MK366369	MK366370	MK366371
A/swine/Garrel/13676/2011 (H1N1)	Germany	25-Jul-2011	MK366372	MK366373	MK366374	MK366375	MK366376	MK366377	MK366378	MK366379
A/swine/Hemmoor/13756/2011 (H1N1)	Germany	15-Aug-2011	MK366380	MK366381	MK366382	MK366383	MK366384	MK366385	MK366386	MK366387

A/swine/Steinfurt/13758/2011 (H1N1)	Germany	14-Aug-2011	MK366388	MK366389	MK366390	MK366391	MK366392	MK366393	MK366394	MK366395
A/swine/Bothenheilingen/13765-3/2011 (H1N1)	Germany	15-Aug-2011	MK366396	MK366397	MK366398	MK366399	MK366400	MK366401	MK366402	MK366403
A/swine/Steinfeld/13785/2011 (H1N1)	Germany	19-Aug-2011	MK366404	MK366405	MK366406	MK366407	MK366408	MK366409	MK366410	MK366411
A/swine/Vechta/13789/2011 (H1N1)	Germany	23-Aug-2011	MK366412	MK366413	MK366414	MK366415	MK366416	MK366417	MK366418	MK366419
A/swine/Heiden/13820/2011 (H1N1)	Germany	24-Aug-2011	MK366420	MK366421	MK366422	MK366423	MK366424	MK366425	MK366426	MK366427
A/swine/Georgsmarienhütte/13824-2/2011 (H1N1)	Germany	29-Aug-2011	MK366428	MK366429	MK366430	MK366431	MK366432	MK366433	MK366434	MK366435
A/swine/Viersen/13836/2011 (H1N1)	Germany	31-Aug-2011	MK366436	MK366437	MK366438	MK366439	MK366440	MK366441	MK366442	MK366443
A/swine/Sittensen/13857-3/2011 (H1N1)	Germany	07-Sep-2011	MK366444	MK366445	MK366446	MK366447	MK366448	MK366449	MK366450	MK366451
A/swine/Ankum/13894/2011 (H1N1)	Germany	12-Sep-2011	MK366452	MK366453	MK366454	MK366455	MK366456	MK366457	MK366458	MK366459
A/swine/Lohne/13895/2011 (H1N1)	Germany	09-Sep-2011	MK366460	MK366461	MK366462	MK366463	MK366464	MK366465	MK366466	MK366467
A/swine/Gossau/14060-1/2011 (H1N1)	Switzerland	14-Oct-2011	MK366468	MK366469	MK366470	MK366471	MK366472	MK366473	MK366474	MK366475
A/swine/Lohne/14087/2011 (H1N1)	Germany	19-Oct-2011	MK366476	MK366477	MK366478	MK366479	MK366480	MK366481	MK366482	MK366483
A/swine/Norden/14100-2/2011 (H1N1)	Germany	20-Oct-2011	MK366484	MK366485	MK366486	MK366487	MK366488	MK366489	MK366490	MK366491
A/swine/Straelen/14101/2011 (H1N1)	Germany	25-Oct-2011	MK366492	MK366493	MK366494	MK366495	MK366496	MK366497	MK366498	MK366499
A/swine/Garrel/14103/2011 (H1N1)	Germany	26-Oct-2011	MK366500	MK366501	MK366502	MK366503	MK366504	MK366505	MK366506	MK366507
A/swine/Ankum/14132/2011 (H1N1)	Germany	02-Nov-2011	MK366508	MK366509	MK366510	MK366511	MK366512	MK366513	MK366514	MK366515
A/swine/Lohne/14134/2011 (H1N1)	Germany	01-Nov-2011	MK366516	MK366517	MK366518	MK366519	MK366520	MK366521	MK366522	MK366523
A/swine/Buch/14162/2011 (H1N1)	Germany	01-Nov-2011	MK366524	MK366525	MK366526	MK366527	MK366528	MK366529	MK366530	MK366531
A/swine/Celle/14169/2011 (H1N1)	Germany	02-Nov-2011	MK366532	MK366533	MK366534	MK366535	MK366536	MK366537	MK366538	MK366539
A/swine/Lohne/14170/2011 (H1N1)	Germany	09-Nov-2011	MK366540	MK366541	MK366542	MK366543	MK366544	MK366545	MK366546	MK366547
A/swine/Friesoythe/14174/2011 (H1N1)	Germany	09-Nov-2011	MK366548	MK366549	MK366550	MK366551	MK366552	MK366553	MK366554	MK366555
A/swine/Heudorf-Meßkirch/IDT14176/2011 (H1N1)	Germany	09-Nov-2011	KC631885	KC631886	KC631887	KC631888	KC631889	KC631890	KC631891	KC631892
A/swine/Neumark/IDT14179/2011 (H1N1)	Germany	10-Nov-2011	KC222585	KC222586	KC222587	KC222588	KC222589	KC222590	KC222591	KC222592
A/swine/Buch/14226-1/2011 (H1N1)	Germany	18-Nov-2011	MK366556	MK366557	MK366558	MK366559	MK366560	MK366561	MK366562	MK366563
A/swine/Hamersen/14228/2011 (H1N1)	Germany	18-Nov-2011	MK366564	MK366565	MK366566	MK366567	MK366568	MK366569	MK366570	MK366571
A/swine/Gronau/14349/2011 (H1N1)	Germany	29-Nov-2011	MK366572	MK366573	MK366574	MK366575	MK366576	MK366577	MK366578	MK366579
A/swine/Wetedorf/14350/2011 (H1N1)	Germany	30-Nov-2011	MK366580	MK366581	MK366582	MK366583	MK366584	MK366585	MK366586	MK366587
A/swine/Pfeffenhausen/14351/2011 (H1N1)	Germany	30-Nov-2011	MK366588	MK366589	MK366590	MK366591	MK366592	MK366593	MK366594	MK366595
A/swine/Ahrenswohlde/14395/2011 (H1N1)	Germany	13-Dec-2011	MK366596	MK366597	MK366598	MK366599	MK366600	MK366601	MK366602	MK366603
A/swine/Büren/14396/2011 (H1N1)	Germany	12-Dec-2011	MK366604	MK366605	MK366606	MK366607	MK366608	MK366609	MK366610	MK366611
A/swine/Bad Wünnenberg/14402/2011 (H1N1)	Germany	08-Dec-2011	MK366612	MK366613	MK366614	MK366615	MK366616	MK366617	MK366618	MK366619
A/swine/Metelen/14413-2/2011 (H1N1)	Germany	14-Dec-2011	MK366620	MK366621	MK366622	MK366623	MK366624	MK366625	MK366626	MK366627
A/swine/Lohne/14425/2011 (H1N1)	Germany	14-Dec-2011	MK366628	MK366629	MK366630	MK366631	MK366632	MK366633	MK366634	MK366635
A/swine/Rheinberg/14457-1/2011 (H1N1)	Germany	14-Dec-2011	MK366636	MK366637	MK366638	MK366639	MK366640	MK366641	MK366642	MK366643
A/swine/Straelen/14460-2/2011 (H1N1)	Germany	22-Dec-2011	MK366644	MK366645	MK366646	MK366647	MK366648	MK366649	MK366650	MK366651
A/swine/Ochtrup/14485-1/2011 (H1N1)	Germany	22-Dec-2011	MK366652	MK366653	MK366654	MK366655	MK366656	MK366657	MK366658	MK366659
A/swine/Ochtrup/14606/2012 (H1N1)	Germany	13-Jan-2012	MK366660	MK366661	MK366662	MK366663	MK366664	MK366665	MK366666	MK366667
A/swine/Lohne/14611/2012 (H1N1)	Germany	19-Jan-2012	MK366668	MK366669	MK366670	MK366671	MK366672	MK366673	MK366674	MK366675
A/swine/Neuharlingersiel/14624-3/2012 (H1N1)	Germany	23-Jan-2012	MK366676	MK366677	MK366678	MK366679	MK366680	MK366681	MK366682	MK366683
A/swine/Ahlen/14631/2012 (H1N1)	Germany	18-Jan-2012	MK366684	MK366685	MK366686	MK366687	MK366688	MK366689	MK366690	MK366691
A/swine/Harsefeld/14637/2012 (H1N1)	Germany	24-Jan-2012	MK366692	MK366693	MK366694	MK366695	MK366696	MK366697	MK366698	MK366699
A/swine/Rheda/14657/2012 (H1N1)	Germany	24-Jan-2012	MK366700	MK366701	MK366702	MK366703	MK366704	MK366705	MK366706	MK366707
A/swine/Billerbeck/14666/2012 (H1N1)	Germany	30-Jan-2012	MK366708	MK366709	MK366710	MK366711	MK366712	MK366713	MK366714	MK366715
A/swine/Rosendahl/14669-3/2012 (H1N1)	Germany	25-Jan-2012	MK366716	MK366717	MK366718	MK366719	MK366720	MK366721	MK366722	MK366723
A/swine/Emsbüren/14689-2/2012 (H1N1)	Germany	31-Jan-2012	MK366724	MK366725	MK366726	MK366727	MK366728	MK366729	MK366730	MK366731
A/swine/Frankfurt/14693/2012 (H1N1)	Germany	01-Feb-2012	MK366732	MK366733	MK366734	MK366735	MK366736	MK366737	MK366738	MK366739
A/swine/Friesoythe/14696/2012 (H1N1)	Germany	01-Feb-2012	MK366740	MK366741	MK366742	MK366743	MK366744	MK366745	MK366746	MK366747
A/swine/Lohne/14721-1/2012 (H1N1)	Germany	06-Feb-2012	MK366748	MK366749	MK366750	MK366751	MK366752	MK366753	MK366754	MK366755
A/swine/Legden/14730/2012 (H1N1)	Germany	09-Feb-2012	MK366756	MK366757	MK366758	MK366759	MK366760	MK366761	MK366762	MK366763
A/swine/Lohne/14766/2012 (H1N1)	Germany	13-Feb-2012	MK366764	MK366765	MK366766	MK366767	MK366768	MK366769	MK366770	MK366771
A/swine/Marsberg/14769/2012 (H1N1)	Germany	14-Feb-2012	MK366772	MK366773	MK366774	MK366775	MK366776	MK366777	MK366778	MK366779
A/swine/Diemelstadt/14770/2012 (H1N1)	Germany	14-Feb-2012	MK366780	MK366781	MK366782	MK366783	MK366784	MK366785	MK366786	MK366787
A/swine/Coesfeld/14811/2012 (H1N1)	Germany	17-Feb-2012	MK366788	MK366789	MK366790	MK366791	MK366792	MK366793	MK366794	MK366795
A/swine/Scharfenberg/15021/2012 (H1N1)	Germany	12-Mar-2012	MK366796	MK366797	MK366798	MK366799	MK366800	MK366801	MK366802	MK366803
A/swine/Ruploh/15022/2012 (H1N1)	Germany	06-Mar-2012	MK366804	MK366805	MK366806	MK366807	MK366808	MK366809	MK366810	MK366811
A/swine/Lüdinghausen/15060-1/2012 (H1N1)	Germany	20-Mar-2012	MK366812	MK366813	MK366814	MK366815	MK366816	MK366817	MK366818	MK366819

A/swine/Dülmen/15075/2012 (H1N1)	Germany	21-Mar-2012	MK366820	MK366821	MK366822	MK366823	MK366824	MK366825	MK366826	MK366827
A/swine/Fuldatal/15116/2012 (H1N1)	Germany	24-Mar-2012	MK366828	MK366829	MK366830	MK366831	MK366832	MK366833	MK366834	MK366835
A/swine/Nordhorn/15118/2012 (H1N1)	Germany	29-Mar-2012	MK366836	MK366837	MK366838	MK366839	MK366840	MK366841	MK366842	MK366843
A/swine/Rottenburg/15122/2012 (H1N1)	Germany	12-Apr-2012	MK366844	MK366845	MK366846	MK366847	MK366848	MK366849	MK366850	MK366851
A/swine/Lohne/15137/2012 (H1N1)	Germany	05-Apr-2012	MK366852	MK366853	MK366854	MK366855	MK366856	MK366857	MK366858	MK366859
A/swine/Drensteinfurt/15193-1/2012 (H1N1)	Germany	16-Apr-2012	MK366860	MK366861	MK366862	MK366863	MK366864	MK366865	MK366866	MK366867
A/swine/Wohnste/15223/2012 (H1N1)	Germany	18-Apr-2012	MK366868	MK366869	MK366870	MK366871	MK366872	MK366873	MK366874	MK366875
A/swine/Lohne/15374/2012 (H1N1)	Germany	21-May-2012	MK366876	MK366877	MK366878	MK366879	MK366880	MK366881	MK366882	MK366883
A/swine/Meppen/15514/2012 (H1N1)	Germany	25-Jun-2012	MK366884	MK366885	MK366886	MK366887	MK366888	MK366889	MK366890	MK366891
A/swine/Neuenhaus/16826/2013 (H1N1)	Germany	07-Jan-2013	MK366892	MK366893	MK366894	MK366895	MK366896	MK366897	MK366898	MK366899
A/swine/Mengkofen/17020/2013 (H1N1)	Germany	14-Jan-2013	MK366900	MK366901	MK366902	MK366903	MK366904	MK366905	MK366906	MK366907
A/swine/Hirschberg/17193/2013(H1N1)	Germany	12-Feb-2013	MK366908	MK366909	MK366910	MK366911	MK366912	MK366913	MK366914	MK366915
A/swine/Wurster Nordseeküste/17217/2013 (H1N1)	Germany	15-Feb-2013	MK366916	MK366917	MK366918	MK366919	MK366920	MK366921	MK366922	MK366923
A/swine/Schneiderkrug/17263/2013 (H1N1)	Germany	22-Feb-2013	MK366924	MK366925	MK366926	MK366927	MK366928	MK366929	MK366930	MK366931
A/swine/Klein Wanzleben/17303/2013 (H1N1)	Germany	26-Feb-2013	MK366932	MK366933	MK366934	MK366935	MK366936	MK366937	MK366938	MK366939
A/swine/Kalefeld/17614/2013 (H1N1)	Germany	26-Mar-2013	MK366940	MK366941	MK366942	MK366943	MK366944	MK366945	MK366946	MK366947
A/swine/Minden-Lübbecke/17934/2013 (H1N1)	Germany	23-May-2013	MK366948	MK366949	MK366950	MK366951	MK366952	MK366953	MK366954	MK366955
A/swine/Melle/18077/2013 (H1N1)	Germany	21-Jun-2013	MK366956	MK366957	MK366958	MK366959	MK366960	MK366961	MK366962	MK366963
A/swine/Ahaus/18137/2013 (H1N1)	Germany	01-Jul-2013	MK366964	MK366965	MK366966	MK366967	MK366968	MK366969	MK366970	MK366971
A/swine/Langenamming/18152/2013 (H1N1)	Germany	01-Jul-2013	MK366972	MK366973	MK366974	MK366975	MK366976	MK366977	MK366978	MK366979
A/swine/Lüdinghausen/18391/2013 (H1N1)	Germany	06-Aug-2013	MK366980	MK366981	MK366982	MK366983	MK366984	MK366985	MK366986	MK366987
A/swine/Ascheberg/18498/2013 (H1N1)	Germany	28-Aug-2013	MK366988	MK366989	MK366990	MK366991	MK366992	MK366993	MK366994	MK366995
A/swine/Ascheberg/18499/2013 (H1N1)	Germany	29-Aug-2013	MK366996	MK366997	MK366998	MK366999	MK367000	MK367001	MK367002	MK367003
A/swine/Nordkirchen/18822/2013 (H1N1)	Germany	02-Oct-2013	MK367004	MK367005	MK367006	MK367007	MK367008	MK367009	MK367010	MK367011
A/swine/Drensteinfurt/19244/2013 (H1N1)	Germany	26-Nov-2013	MK367012	MK367013	MK367014	MK367015	MK367016	MK367017	MK367018	MK367019
A/swine/Drensteinfurt/19376/2013 (H1N1)	Germany	11-Dec-2013	MK367020	MK367021	MK367022	MK367023	MK367024	MK367025	MK367026	MK367027
A/swine/Sandbeiendorf/19533/2014 (H1N1)	Germany	21-Jan-2014	MK367028	MK367029	MK367030	MK367031	MK367032	MK367033	MK367034	MK367035
A/swine/Erwitte/19661/2014 (H1N1)	Germany	04-Feb-2014	MK367036	MK367037	MK367038	MK367039	MK367040	MK367041	MK367042	MK367043
A/swine/Bothenheilingen/19699/2014 (H1N1)	Germany	17-Feb-2014	MK367044	MK367045	MK367046	MK367047	MK367048	MK367049	MK367050	MK367051
A/swine/Bad Griesbach/19758/2014 (H1N1)	Germany	24-Feb-2014	MK367052	MK367053	MK367054	MK367055	MK367056	MK367057	MK367058	MK367059
A/swine/Borken/19808/2014 (H1N1)	Germany	05-Mar-2014	MK367060	MK367061	MK367062	MK367063	MK367064	MK367065	MK367066	MK367067
A/swine/Rügheim/19841/2014 (H1N1)	Germany	06-Mar-2014	MK367068	MK367069	MK367070	MK367071	MK367072	MK367073	MK367074	MK367075
A/swine/Bockhorn/19911/2014 (H1N1)	Germany	24-Mar-2014	MK367076	MK367077	MK367078	MK367079	MK367080	MK367081	MK367082	MK367083
A/swine/Ahorn/19945/2014 (H1N1)	Germany	31-Mar-2014	MK367084	MK367085	MK367086	MK367087	MK367088	MK367089	MK367090	MK367091
A/swine/Schallern/IDT19989/2014 (H1N1)	Germany	11-Apr-2014	KX013010	KX013011	KX013012	KX013013	KX013014	KX013015	KX013016	KX013017
A/swine/Ascheberg/19992/2014 (H1N1)	Germany	11-Apr-2014	MK367092	MK367093	MK367094	MK367095	MK367096	MK367097	MK367098	MK367099
A/swine/Mörsbrunn/20096/2014 (H1N1)	Germany	06-May-2014	MK367100	MK367101	MK367102	MK367103	MK367104	MK367105	MK367106	MK367107
A/swine/Essen/20452/2014 (H1N1)	Germany	31-Jul-2014	MK367108	MK367109	MK367110	MK367111	MK367112	MK367113	MK367114	MK356115
A/swine/Bad Wünnenberg/20518/2014 (H1N1)	Germany	20-Aug-2014	MK367116	MK367117	MK367118	MK367119	MK367120	MK367121	MK367122	MK367123
A/swine/Rosendahl/20634/2014 (H1N1)	Germany	16-Sep-2014	MK367124	MK367125	MK367126	MK367127	MK367128	MK367129	MK367130	MK367131
A/swine/Everswinkel/20648/2014 (H1N1)	Germany	12-Sep-2014	MK367132	MK367133	MK367134	MK367135	MK367136	MK367137	MK367138	MK367139
A/swine/Wachtum/20657/2014 (H1N1)	Germany	10-Aug-2014	MK367140	MK367141	MK367142	MK367143	MK367144	MK367145	MK367146	MK367147
A/swine/Lohne/20709/2014 (H1N1)	Germany	06-Oct-2014	MK367148	MK367149	MK367150	MK367151	MK367152	MK367153	MK367154	MK367155
A/swine/Garrel/20734/2014 (H1N1)	Germany	10-Oct-2014	MK367156	MK367157	MK367158	MK367159	MK367160	MK367161	MK367162	MK367163
A/swine/Baarle Nassau/20825/2014 (H1N1)	Austria	26-Oct-2014	MK367164	MK367165	MK367166	MK367167	MK367168	MK367169	MK367170	MK367171
A/swine/Feldbach/20834/2014 (H1N1)	Austria	23-Oct-2014	MK367172	MK367173	MK367174	MK367175	MK367176	MK367177	MK367178	MK367179
A/swine/Rüthen/20843/2014 (H1N1)	Germany	24-Oct-2014	MK367180	MK367181	MK367182	MK367183	MK367184	MK367185	MK367186	MK367187
A/swine/Haaren/20977/2014 (H1N1)	Germany	21-Oct-2014	MK367188	MK367189	MK367190	MK367191	MK367192	MK367193	MK367194	MK367195
A/swine/Langen/20991/2014 (H1N1)	Germany	24-Nov-2014	MK367196	MK367197	MK367198	MK367199	MK367200	MK367201	MK367202	MK367203
A/swine/Glabbeek/21006/2014 (H1N1)	Belgium	26-Nov-2014	MK367204	MK367205	MK367206	MK367207	MK367208	MK367209	MK367210	MK367211
A/swine/Steinfeld/21010/2014 (H1N1)	Germany	25-Nov-2014	MK367212	MK367213	MK367214	MK367215	MK367216	MK367217	MK367218	MK367219
A/swine/Mariaheide/21031/2014 (H1N1)	Netherlands	27-Nov-2014	MK367220	MK367221	MK367222	MK367223	MK367224	MK367225	MK367226	MK367227
A/swine/Harreveld/21082/2014 (H1N1)	Netherlands	11-Dec-2014	MK367228	MK367229	MK367230	MK367231	MK367232	MK367233	MK367234	MK367235
A/swine/Borken/21107/2014 (H1N1)	Germany	10-Dec-2014	MK367236	MK367237	MK367238	MK367239	MK367240	MK367241	MK367242	MK367243
A/swine/Heinsberg/21114/2014 (H1N1)	Germany	22-Dec-2014	MK367244	MK367245	MK367246	MK367247	MK367248	MK367249	MK367250	MK367251
A/swine/Werl/21120/2014 (H1N1)	Germany	22-Dec-2014	MK367252	MK367253	MK367254	MK367255	MK367256	MK367257	MK367258	MK367259

A/swine/Moenchengladbach/21124/2015 (H1N1)	Germany	05-Jan-2015	MK367260	MK367261	MK367262	MK367263	MK367264	MK367265	MK367266	MK367267
A/swine/Oldenzaal/21126/2014 (H1N1)	Netherlands	16-Dec-2014	MK367268	MK367269	MK367270	MK367271	MK367272	MK367273	MK367274	MK367275
A/swine/Cloppenburg/21149/2014 (H1N1)	Germany	21-Dec-2014	MK367276	MK367277	MK367278	MK367279	MK367280	MK367281	MK367282	MK367283
A/swine/Rhede/21178/2015 (H1N1)	Germany	16-Jan-2015	MK367284	MK367285	MK367286	MK367287	MK367288	MK367289	MK367290	MK367291
A/swine/Goch/21216/2015 (H1N1)	Germany	22-Jan-2015	MK367292	MK367293	MK367294	MK367295	MK367296	MK367297	MK367298	MK367299
A/swine/Cloppenburg/21225/2015 (H1N1)	Germany	26-Jan-2015	MK367300	MK367301	MK367302	MK367303	MK367304	MK367305	MK367306	MK367307
A/swine/Cloppenburg/21226/2015 (H1N1)	Germany	26-Jan-2015	MK367308	MK367309	MK367310	MK367311	MK367312	MK367313	MK367314	MK367315
A/swine/Graben/21396-2/2015 (H1N1)	Germany	02-Mar-2015	MK367316	MK367317	MK367318	MK367319	MK367320	MK367321	MK367322	MK367323
A/swine/Oirlo/21422/2015 (H1N1)	Netherlands	10-Mar-2015	MK367324	MK367325	MK367326	MK367327	MK367328	MK367329	MK367330	MK367331
A/swine/Steinberg/21495/2015 (H1N1)	Germany	25-Mar-2015	MK367332	MK367333	MK367334	MK367335	MK367336	MK367337	MK367338	MK367339
A/swine/Diepholz/21603/2015 (H1N1)	Germany	15-Apr-2015	MK367340	MK367341	MK367342	MK367343	MK367344	MK367345	MK367346	MK367347
A/swine/Schwedeneck/21724/2015 (H1N1)	Germany	21-May-2015	MK367348	MK367349	MK367350	MK367351	MK367352	MK367353	MK367354	MK367355
A/swine/Lüdinghausen/21728/2015 (H1N1)	Germany	21-May-2015	MK367356	MK367357	MK367358	MK367359	MK367360	MK367361	MK367362	MK367363
A/swine/Soest/21748/2015 (H1N1)	Germany	26-May-2015	MK367364	MK367365	MK367366	MK367367	MK367368	MK368369	MK367370	MK367371
A/swine/Sögel/22071/2015 (H1N1)	Germany	26-Aug-2015	MK367372	MK367373	MK367374	MK367375	MK367376	MK367377	MK367378	MK367379
A/swine/Dülmen/22335/2015 (H1N1)	Germany	29-Oct-2015	MK367380	MK367381	MK367382	MK367383	MK367384	MK367385	MK367386	MK367387
