# Supplementary figures and images for: Cocirculation of Swine H1N1 Influenza A Virus Lineages in Germany
Source: Viruses. 2020 Jul 15;12(7):762. doi: 10.3390/v12070762 (PMC7411773; doi:10.3390/v12070762)

Suppl. Fig. 1

HAH1

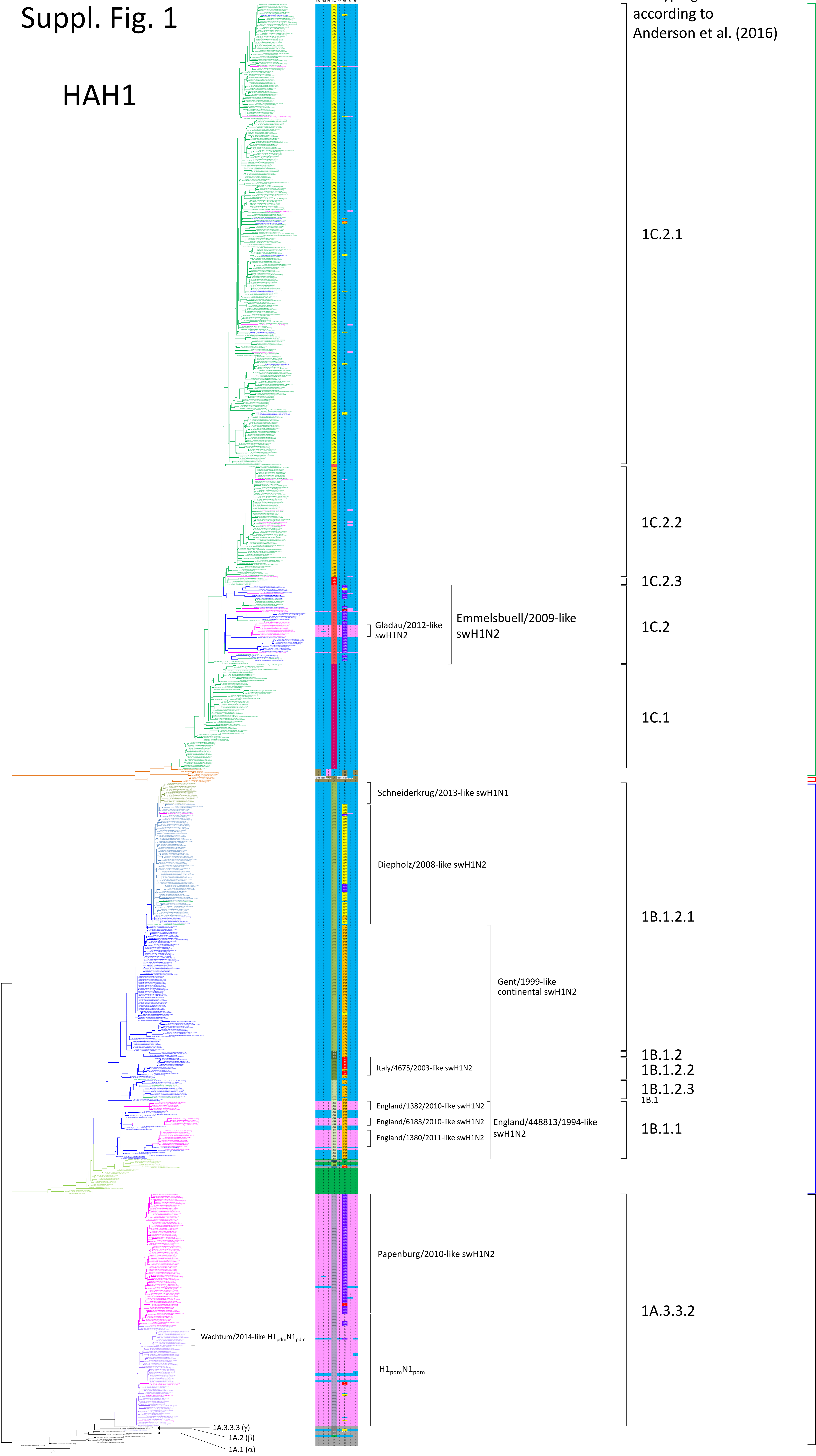

Lineage  
1C

Avian lineages

Lineage  
1B

Lineage  
1A

Supplement: Supplementary file 1 [file viruses-12-00762-s001.zip › Zell_et_al_Supplementary_Files_revised/Fig S1.pdf]

Suppl. Fig. 3

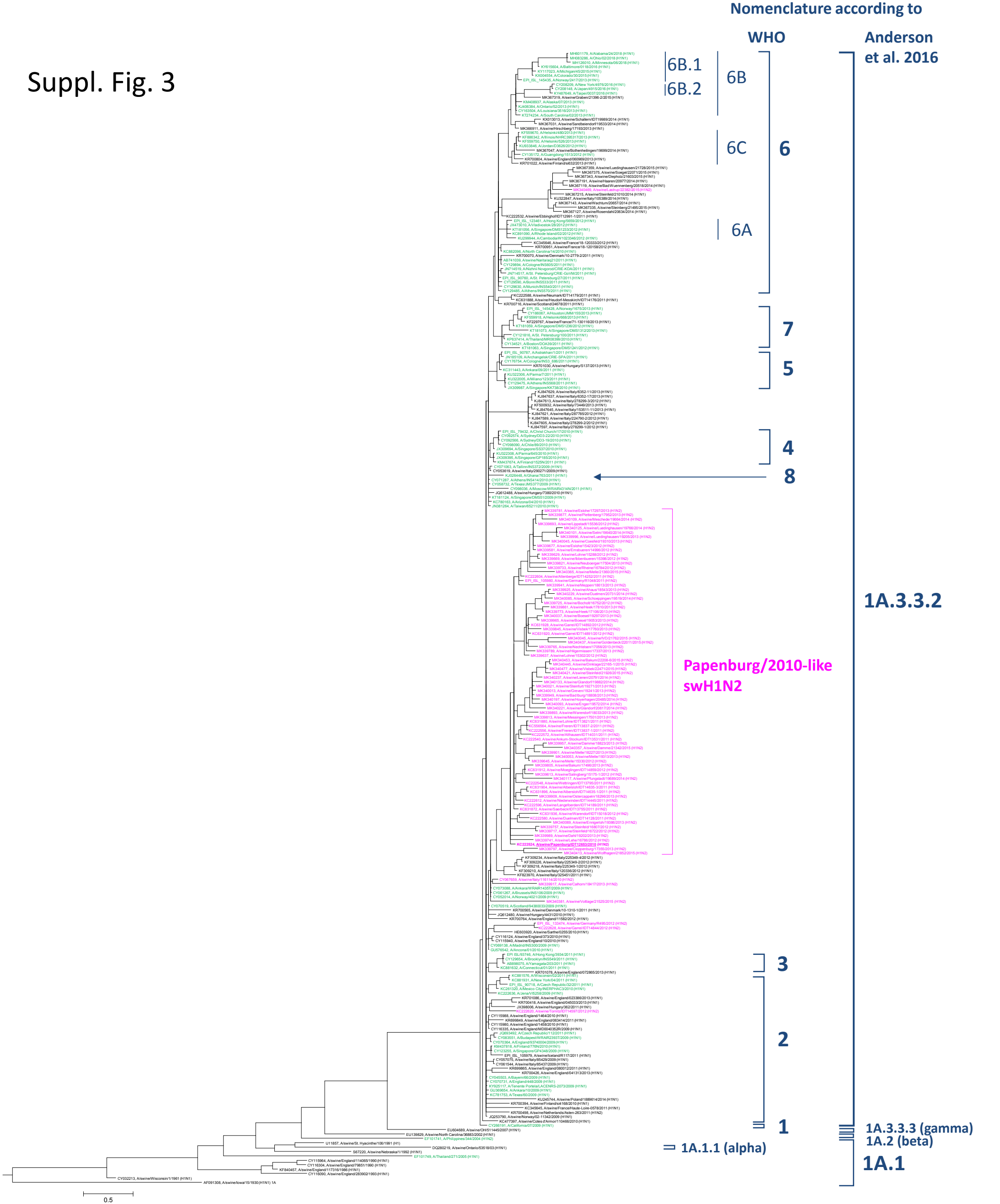

Supplement: Supplementary file 1 [file viruses-12-00762-s001.zip › Zell_et_al_Supplementary_Files_revised/Fig S3.pdf]

Suppl. Fig. 4

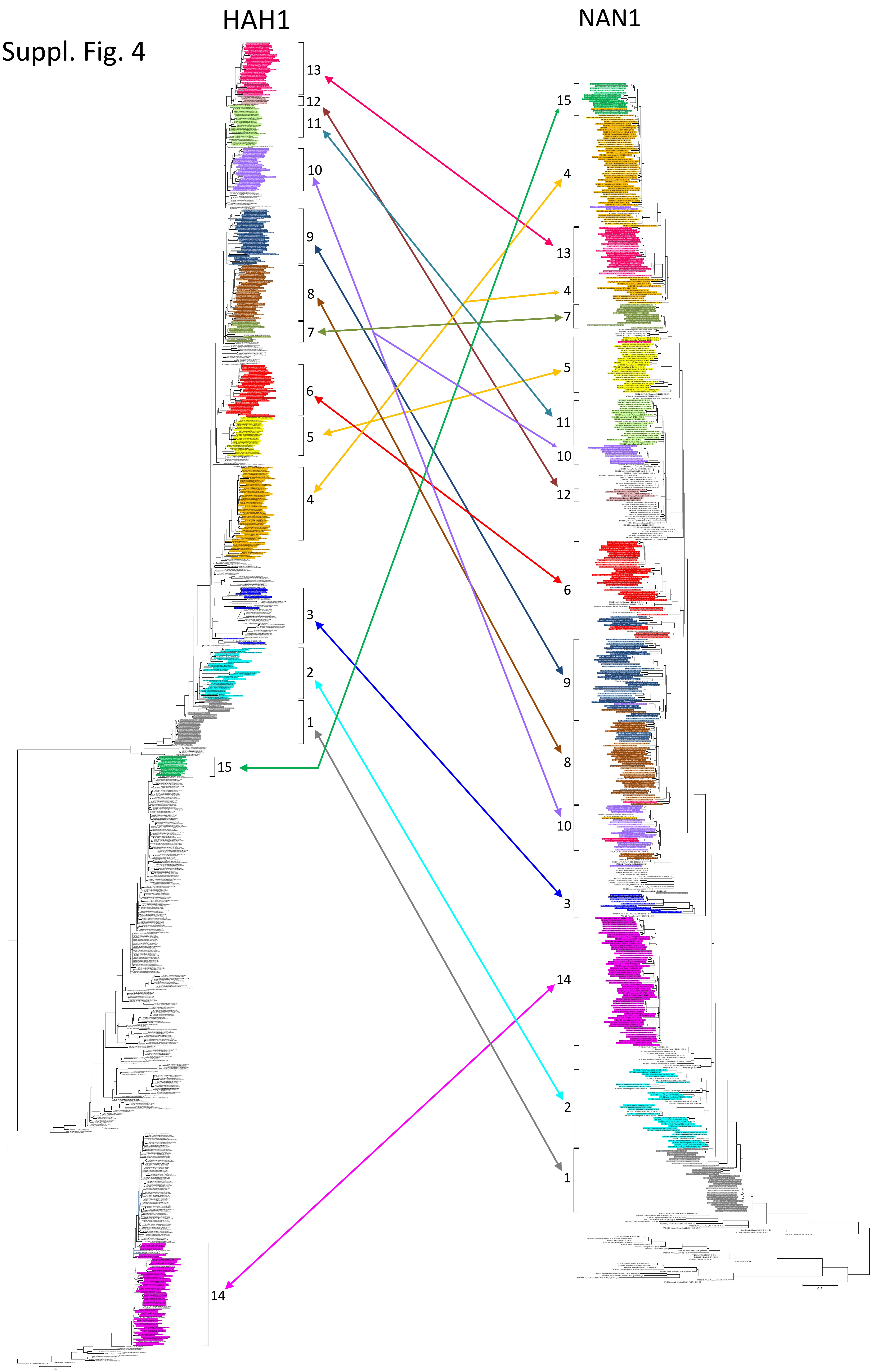

Supplement: Supplementary file 1 [file viruses-12-00762-s001.zip › Zell_et_al_Supplementary_Files_revised/Fig S4.pdf]

Suppl. Fig. 5A

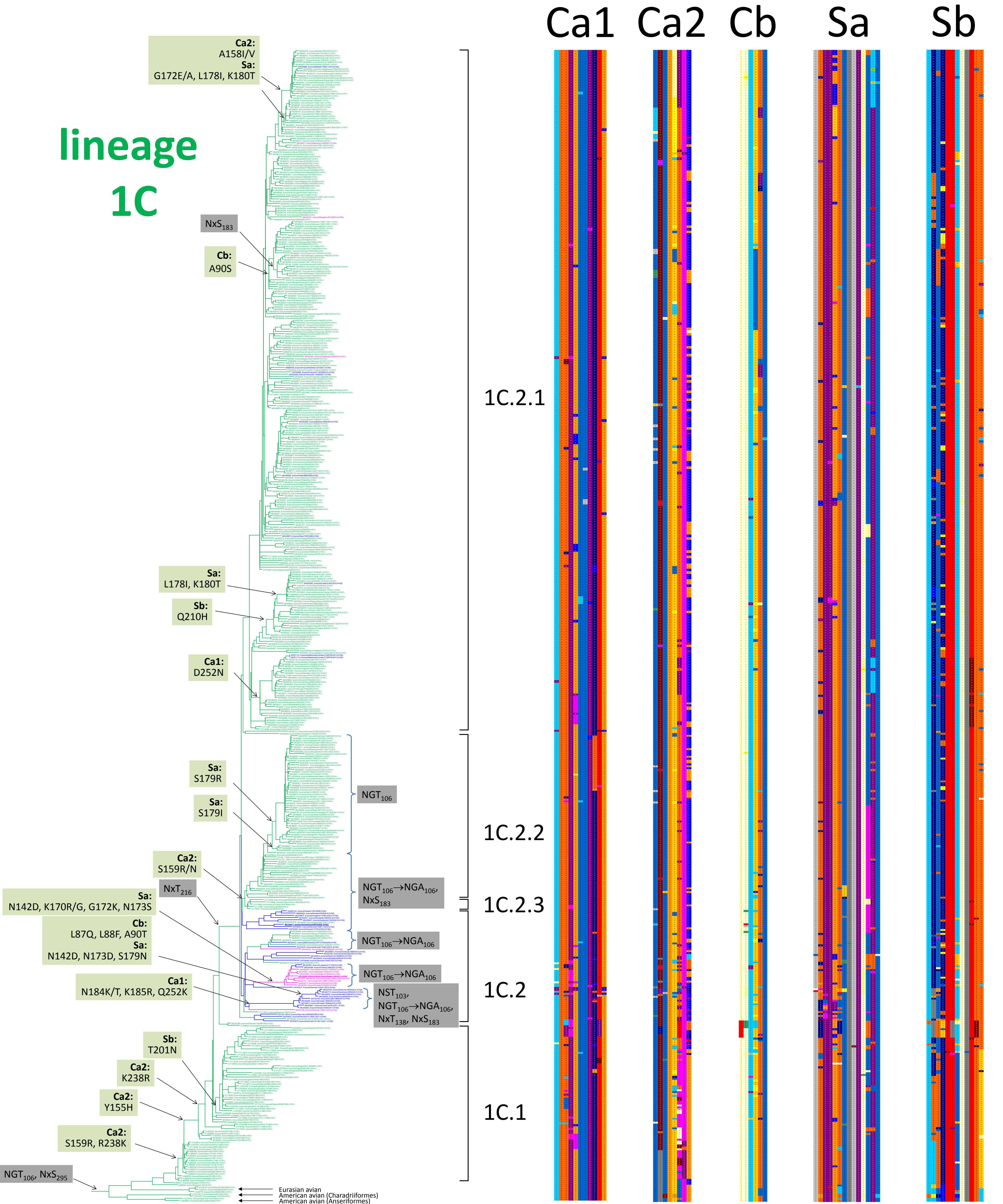

Supplement: Supplementary file 1 [file viruses-12-00762-s001.zip › Zell_et_al_Supplementary_Files_revised/Fig S5A.pdf]

# Suppl. Fig. 5B

**lineage**  
**1B**

Ca1

Ca<sup>2+</sup>

**Cb**

Sa

Sb

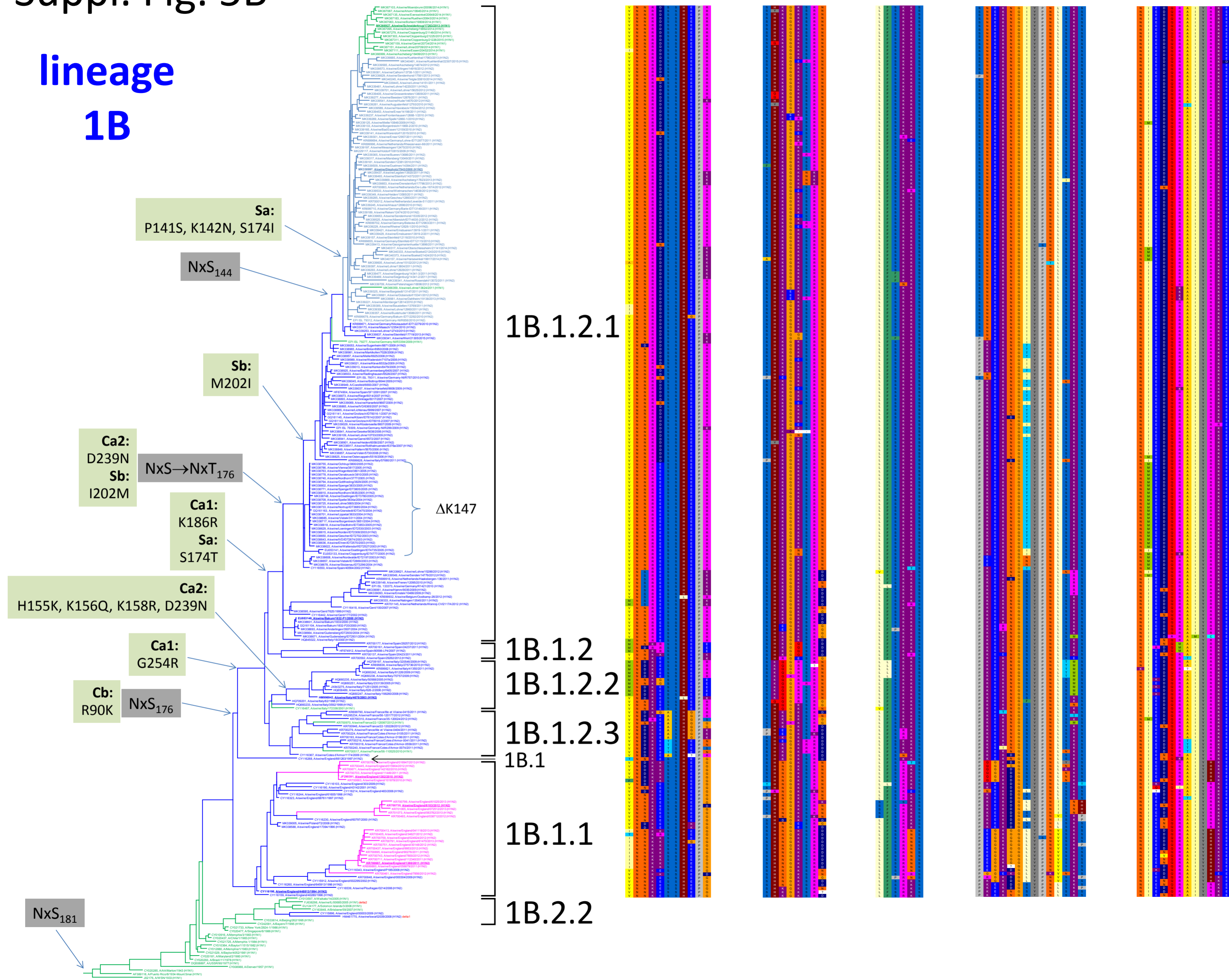

Supplement: Supplementary file 1 [file viruses-12-00762-s001.zip › Zell_et_al_Supplementary_Files_revised/Fig S5B.pdf]
